# Identification of motifs that function in the splicing of non-canonical introns

**DOI:** 10.1186/gb-2008-9-6-r97

**Published:** 2008-06-12

**Authors:** Jill I Murray, Rodger B Voelker, Kristy L Henscheid, M Bryan Warf, J Andrew Berglund

**Affiliations:** 1 Institute of Molecular Biology and Department of Chemistry, University of Oregon, Eugene, Oregon, USA

## Abstract

**Background:**

While the current model of pre-mRNA splicing is based on the recognition of four canonical intronic motifs (5' splice site, branchpoint sequence, polypyrimidine (PY) tract and 3' splice site), it is becoming increasingly clear that splicing is regulated by both canonical and non-canonical splicing signals located in the RNA sequence of introns and exons that act to recruit the spliceosome and associated splicing factors. The diversity of human intronic sequences suggests the existence of novel recognition pathways for non-canonical introns. This study addresses the recognition and splicing of human introns that lack a canonical PY tract. The PY tract is a uridine-rich region at the 3' end of introns that acts as a binding site for U2AF65, a key factor in splicing machinery recruitment.

**Results:**

Human introns were classified computationally into low- and high-scoring PY tracts by scoring the likely U2AF65 binding site strength. Biochemical studies confirmed that low-scoring PY tracts are weak U2AF65 binding sites while high-scoring PY tracts are strong U2AF65 binding sites. A large population of human introns contains weak PY tracts. Computational analysis revealed many families of motifs, including C-rich and G-rich motifs, that are enriched upstream of weak PY tracts. *In vivo *splicing studies show that C-rich and G-rich motifs function as intronic splicing enhancers in a combinatorial manner to compensate for weak PY tracts.

**Conclusion:**

The enrichment of specific intronic splicing enhancers upstream of weak PY tracts suggests that a novel mechanism for intron recognition exists, which compensates for a weakened canonical pre-mRNA splicing motif.

## Abstract 2

The enrichment of specific intronic splicing enhancers upstream of weak PY tracts suggests a novel mechanism for intron recognition that compensates for a weakened canonical pre-mRNA splicing motif.

## Background

Pre-mRNA splicing is an essential processing step where non-coding intervening sequences (introns) are removed from the initial RNA transcript and coding sequences (exons) are ligated together to produce mature mRNA. Pre-mRNA splicing is mediated by the spliceosome, a multi-component complex composed of small nuclear ribonucleoproteins (snRNPs) and over 100 accessory proteins [1]. The splicing machinery assembles on the pre-mRNA in a highly regulated fashion to carry out the process of removing the intron and ligating the two adjoining exons [2,3]. Pre-mRNA splicing relies on the accurate recognition of the splice junctions that define introns and exons. This is underlined by the observation that incorrect pre-mRNA splicing is a major contributor to human genetic diseases [4-6]. Not only is splicing a crucial step in the accurate transfer of genetic information from DNA to RNA to protein, it is also a step that allows for regulation of gene expression as well as increased protein diversity through alternative splicing decisions [7].

Several canonical intronic sequences define an intron and recruit the spliceosome to the pre-mRNA: the 5' splice site (5'ss, AG/GURAGU), the branchpoint sequence (CURAY), the polypyrimidine (PY) tract (a run of polypyrimidines located between the 3' splice site and the branchpoint), and the 3' splice site (3'ss, YAG). These four canonical intronic sequences are recognized by specific components of the spliceosome or associated splicing factors. In the initial stage of splicing, when the decision to remove an intron is made, the U1 snRNP recognizes the 5'ss [8,9], splicing factor 1 (SF1, also known as BBP) recognizes the branchpoint sequence [10,11], and U2AF65 (U2AF (U2 snRNP auxillary factor), 65 kDa subunit) recognizes the PY tract [12,13] while its heterodimer partner U2AF35 (U2AF 35 kDa subunit) recognizes the 3'ss [14-16]. After these initial recognition events, U2AF65 interacts with the U2 snRNP in order to recruit it to the branchpoint sequence, where it displaces SF1 [17,18].

Although canonical splice elements are located within the intron, the exon is generally considered to be the unit that is first recognized and defined by the spliceosome. This is known as exon definition and is thought to be a dominant mode of recognition in human genes where the exons are small and the introns are large [19]. In the exon definition model, the exon and flanking upstream and downstream splice junctions are recognized and bridging interactions across the exon are important for accurate splicing. Conversely, according to the intron definition model, the splice junctions within the intron are recognized and bridging interactions across the intron mediate accurate splicing [19,20]. Intron definition is proposed to be the dominant mode of recognition for small introns [19].

It has become clear that the four canonical splice elements do not contain adequate sequence information to ensure accurate splicing [3]. Additional *cis*-elements appear to be essential for accurate identification of many splice sites, and various *cis*-splicing elements have been identified in both exonic and intronic regions. Based upon their locations and effects upon splicing, these have been categorized as exonic and intronic splicing enhancers (ESEs and ISEs, respectively) or exonic and intronic splicing silencers (ESSs and ISSs, respectively) (for reviews see [21-26]).

We are interested in the question of how introns that lack a canonical splice element are recognized and spliced. We have focused on introns that lack a canonical PY tract. In humans, U2AF65 binding to the PY tract is believed to be critical for intron recognition and splicing. *In vitro *selection studies have determined that U2AF65 binds with highest affinity to continuous runs of uridines interrupted by cytidines [27]. This agrees with the general observation that good PY tracts contain runs of uridines. We have observed that many human introns lack these canonical PY tracts. This leads to the question of how introns lacking strong U2AF65 binding sites are recognized and are able to recruit the U2 snRNP.

One model predicts that U2AF65 is not essential for the splicing of these introns. Several human introns have been shown to be spliced when U2AF65 levels are significantly reduced by RNA interference [28]. U2AF65 may not be required because another splicing factor is functioning to recognize the PY tract region. For example, PUF60 has been shown to substitute for U2AF65 *in vitro *for some substrates [29]. There is the potential that other, yet unidentified, U2AF65-like proteins may function to promote 3'ss selection of non-canonical PY tracts. In a second model, U2AF65 is required for splicing but strong U2AF65-PY tract interactions are not. It has recently been observed in fission yeast that introns lacking PY tracts require U2AF for splicing *in vivo *[30]. Alternative pathways for U2AF65 recruitment may function in introns lacking strong PY tracts. For example, additional *cis*-elements present in the intron could alleviate the need for strong U2AF65-RNA interactions. These *cis*-elements could include the branchpoint sequence and 3'ss, which recruit SF1 and U2AF35, respectively, both of which can bind U2AF65 cooperatively through protein-protein interactions [11,31,32]. Auxiliary *cis*-elements such as ESEs and ISEs could function in the recognition of introns containing weak PY tracts. Previous studies have indicated that ESEs located in the downstream exon are able to compensate for weak PY tracts [33,34]. In this model, the ESEs are recognized by SR (serine/arginine-rich) proteins that interact with the U2AF65/35 heterodimer to help recruit U2AF65 to the 3' end of the intron [34-36]. We propose that a similar mechanism exists where ISEs in the region upstream of the PY tract function to compensate for weak U2AF65 binding by helping to recruit either U2AF65 or U2AF65-recruiting proteins or bypassing the need for U2AF65 in recruiting the U2 snRNP to the intron.

We have used a computational approach to classify human introns in terms of their U2AF65 binding site strength. We conclude that a significant population of human introns does not contain a strong U2AF65 binding site in the PY tract region. This classification of human PY tract strength enabled us to computationally identify intronic motifs over-represented upstream of weak PY tracts. We propose that these over-represented motifs are putative ISEs that are important for the splicing of introns containing weak PY tracts.

LCAT (lecithin cholesterol acyltransferase) intron 4 is a short (83 nucleotide) constitutively spliced intron with a weak PY tract. Mutation of the branchpoint sequence U to C (CUGAC), is known to result in intron retention, causing familial LCAT deficiency (complete deficiency) or fish-eye disease (partial deficiency), which can lead to premature atherosclerosis [37]. Intron retention, rather than skipping, suggests an intron definition model of recognition [19]. Therefore, we expected that ISEs might be involved in the recognition of this intron. We present results showing that G-rich and C-rich motifs, similar to those predicted by our computational approach to be enriched upstream of weak PY tracts, are ISEs important for the splicing of LCAT intron 4, which has a weak PY tract. Furthermore, we have observed that the G-rich and C-rich ISEs function in a combinatorial manner to promote the recognition of a weak PY tract-containing intron. Finally, we show another example of an intron, GNPTG (N-acetylglucosamine-1-phosphotransferase gamma subunit) intron 2, in which C-rich ISEs again appear to be compensating for a weak PY tract.

## Results

### Computational analysis of human intron PY tracts using a U2AF65 binding site scoring method

U2AF65 plays an important role during splicing and is known to bind to the PY tract region located between the branchpoint sequence and the acceptor splice junction [38]. Visual inspection of human introns reveals that, although the PY tract region is enriched in uridines in general, there is a great deal of sequence variation between introns. This degeneracy, at least in part, appears to reflect the low RNA site specificity that U2A65 displays compared to other RNA binding proteins that evolved to recognize highly specific targets. U2AF65 binds with high affinity to contiguous runs of uridines but appears to tolerate moderate interruptions of other nucleotides [27,39-41]. Despite the ability of U2AF65 to bind to degenerate sites, an effective binding site must still be composed primarily of uridines [40,41]. However, many thousands of human introns contain PY tracts that do not contain any sequences that are likely to be effective binding sites (shown below). Many of these PY tracts either contain contiguous runs of cytidines or contain numerous purines, neither of which are likely to represent binding sites for U2AF65 [40,41]. Therefore, it is likely that individual human intronic PY tracts possess a wide range of affinities towards U2AF65, and that many may possess only weak binding sites for it. It is possible that additional *cis*-sequence elements augment the role of the PY tract during splicing, and that such elements play crucial roles in splicing in the absence of a strong U2AF65 binding site.

**Figure 1:**
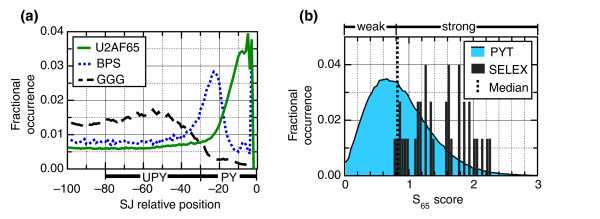
Computational analysis of human intron PY tracts. **(a) **Distribution of intronic motifs (branchpoint (BPS), G-triples (GGG) and U2AF65 binding sites (U2AF65)) adjacent to the 3' end of human introns. The BPS curve is a composite of the distribution of all pentamers containing YTRAC (Y = T or C, R = A or G). The G-triple curve is the composite for all pentamers containing GGG. The U2AF65 curve is a composite of the occurrence of the ten most abundant pentamers found in the U2AF65 SELEX sequences [27,39] (Additional data file 1). The distributions were determined over all human introns, and for each curve the total area under the curve was normalized to unity. The two regions used in this study are depicted below the curves. The PY tract region consisted of the region from -30 to -3, and the upstream PY (UPY) tract region was defined to be from -80 to -30 (relative to the acceptor splice-junction (SJ)). **(b) **Distribution of U2AF65 binding site scores (S_65 _scores) for all human introns (filled blue) and for the U2AF65 SELEX sequences used as the training set for the binding site score (vertical solid black lines). The distributions were generated using a bin size of 0.02, and the total area under the curves was normalized to unity. The median (used as the cutoff for 'weak' and 'strong' binding sites) is depicted as a vertical dashed line.

Many human introns have been shown to be enriched in motifs containing GGG in the region upstream of the PY tract [42,43] (Figure 1a). This observation demonstrates that this region is under compositional selection. G-triples located upstream of a weak PY tract have been shown to affect splice site usage [20]. We hypothesized other *cis*-elements may also be located upstream of the PY tract and may compensate for PY tracts containing weak U2AF65 binding sites. To explore this possibility we performed a computational analysis to determine if the region upstream of the PY tract is enriched in specific motifs when the PY tract does not contain a strong U2AF65 binding site.

In order to carry out this analysis, we first needed to correlate the composition of the PY tract of introns with likely affinities towards U2AF65. Several theoretical models have been presented that describe the relationship between binding site composition and the ΔG of binding between nucleic acids and nucleic acid binding proteins [44,45]. These models require the use of a positional frequency model representing the preferred binding site. *In vitro *selection (SELEX) experiments using human U2AF65 did not reveal a well defined consensus motif shared by high affinity RNAs [27,39]. Several computational methods have been developed to define a degenerate consensus motif from a population of sequences that are thought to contain a common, but unknown, motif [46,47]. Though such methods have proven useful, each has its own weaknesses, and all such predictive methods introduce an added level of uncertainty. We decided to develop a computational method to predict the affinity between a short RNA sequence and U2AF65 that is independent of knowledge of a particular consensus binding motif. We refer to this score as an S_65 _score. The S_65_score, for a given intron, is the average degree to which all pentamers (using a sliding window) found in the PY tract region (-30 to -3 relative to the acceptor splice-junction) are themselves enriched within the SELEX derived sequences (see Materials and methods for a complete description).

For this analysis, the PY tract was defined as the region from -30 to -3 (relative to the acceptor splice junction). This region is highly enriched in the pentamers that are most abundant within the U2AF65 selected sequences (Figure 1a and data not shown). Although a small number of introns are thought to possess functional U2AF65 binding sites upstream of this region [48], the general enrichment for uridines in this region (Figure 1a) is consistent with the premise that the bulk of U2AF65 functional binding sites are located adjacent to the acceptor splice-junction.

The S_65 _scores for the SELEX RNAs appear to be normally distributed with a mean of 1.5 (Figure 1b). In contrast, the S_65 _scores for human PY tracts display a slightly skewed distribution with a mean of 0.877 and a median of 0.811. These are shifted significantly to the left (that is, weaker) relative to the scores for the U2AF65 selected RNAs, suggesting that a large portion of human PY tracts represent weaker than optimal U2AF65 binding sites.

We chose to classify PY tracts that score below the median of 0.811 as 'weak' PY tracts and those above 0.811 as 'strong' PY tracts or likely to have high affinity U2AF65 binding sites. Using this designation, only a single SELEX-derived sequence scores as 'weak'. We are therefore asking whether there are statistically significant differences in the composition of the -80 to -30 region of two types of introns: ones that contain a PY tract with affinities similar to those derived using SELEX, and those with PY tracts with lower affinities.

### Binding of U2AF65 to low-scoring PY tracts

**Figure 2:**
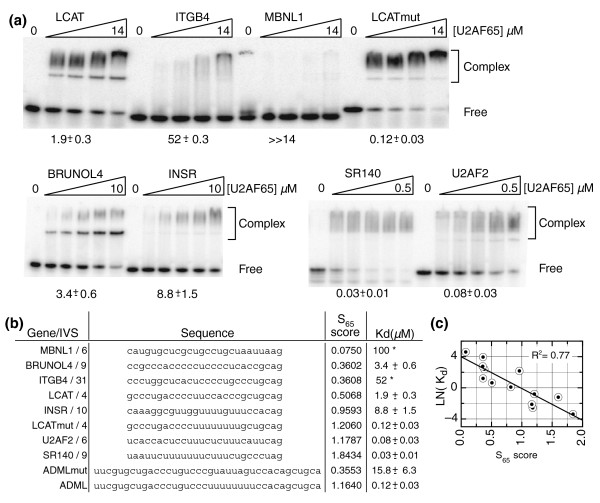
Binding of U2AF65 to human PY tracts validates the U2AF65 SELEX scoring system. **(a) **Gel shift of human U2AF65 with human PY tract RNA oligonucleotides. **(b) **RNA sequences used for binding studies. The gene and intron (IVS) of origin are indicated. The K_d _values are the average of triplicate experiments. K_d _values marked with an asterisk are estimated since the levels of protein required to reach saturation exceed the capacity of the experiment. **(c) **Linear regression of the observed U2AF65 affinities versus the predicted S_65 _score.

In order to asses the relationship between the S_65 _score and observed U2AF65 binding affinities, we evaluated the binding of recombinant human U2AF65 to several human PY tracts of varying S_65 _scores using gel-shift mobility assays (Figure 2). We chose one PY tract that had a very low score (MBNL1 intron 6, S_65 _= 0.0750). This PY tract is interrupted by several purines that are expected to impair U2AF65 binding. We also evaluated three other low-scoring PY tracts with scores closer to the median, and, therefore, correspond to the more 'typical' human PY tract: BRUNOL4 intron 9 (S_65 _= 0.3602), ITGB4 intron 31 (S_65 _= 0.3608), and LCAT intron 4 (S_65 _= 0.5068). All three of these are cytidine-enriched. In addition, we tested three high-scoring PY tracts that had scores spanning the higher range of the distribution: INSR intron 10 (S_65 _= 0.9593), U2AF2 intron 6, (S_65 _= 1.1787), and SR140 intron 9 (S_65 _= 1.8434), and an altered version of the LCAT intron 4 in which the central region was modified to contain an eight nucleotide poly-uridine run (LCATmut with a S_65 _of 1.2060). All four of these high-scoring sequences are uridine-enriched. Binding data were also obtained using two sequences derived from the PY tract of the adenovirus major late (ADML) pre-mRNA, similar to previously studied ADML PY tracts [32,49].

We expected the MBNL1 intron 6 PY tract to represent the weakest U2AF65 binding target and observed no detectable levels of U2AF65 binding at the protein concentrations tested (Figure 2). Meanwhile, all three of the cytidine-rich sequences with moderate S_65 _scores demonstrated moderate affinities in the binding assay. In contrast, three of the uridine-rich sequences (with high S_65 _scores) bound with high affinity. An interesting exception was the INSR-derived sequence, which bound U2AF65 more weakly than the more cytidine-rich LCAT-derived sequence. Importantly, for both LCAT and ADML, the binding of the mutant versions correlates well with the predicted affinities based upon the S_65 _score.

Overall, there is a good agreement between the observed binding affinities for U2AF65 and the predicted affinities based upon the S_65 _score. Plotting the observed K_d _values versus the predicted S_65 _score revealed that the ln of the K_d _appears to be linearly related to the S_65 _score (Figure 2c). Since ΔG is related to K_d _according to the equation Δ*G*° = -*RT*ln(*K*_*d*_), this is consistent with the supposition that S_65 _is linearly related to ΔG. Linear regression of the observed affinities and S_65 _scores demonstrates that these values are strongly correlated (R^2 ^= 0.77; Figure 2c). Some of the observed deviations may be due to influences of RNA secondary structures present in some of the templates. Such secondary structure could greatly influence U2AF65 interactions, but this parameter is not addressed in the S_65 _score. Since U2AF65 is known to have a strong preference for uridines, it is possible that the observed binding affinities simply reflect overall uridine content. However, linear regression analysis of the uridine content versus binding affinities demonstrates that these values are not well correlated (R^2 ^= 0.27, data not shown). Therefore, the S_65 _score is a better predictor of binding affinity than uridine content alone and suggests that U2AF65 is recognizing sequence features more complex than the simple presence or absence of contiguous runs of uridines.

### Introns containing weak PY tracts are enriched in specific motifs upstream of the PY tract

It is possible that introns containing weak U2AF65 binding sites might be enriched in specific sequences that can compensate for the lack of a well-defined PY tract. In order to identify such motifs, we first characterized the relative enrichment of all 4-7 nucleotide n-mers in the 50 nucleotide region from -80 to -30 (relative to the splice-junction) for introns with PY tracts categorized as 'weak' relative to the set of all introns (S_65 _scores less than 0.811; see Materials and methods). We were specifically interested in identifying sequences located in the region upstream of the branchpoint itself. Since most branchpoints are located between -17 and -30 (Figure 1a), the region evaluated would exclude the majority of branchpoint-like sequences.

Human introns have been shown to fall into two classes based upon GC or AT content [50]. In order to be sure that we were not merely measuring compositional biases between AT-rich and GC-rich introns, we classified introns according to the GC content of the last 100 bases. Introns with greater than 50% GC content were categorized as GC-rich while those with less than 50% GC were categorized as AT-rich. As measured using our criteria, 37% of AT-rich introns were found to have 'weak' PY tracts, and 72% of GC-rich introns were determined to have 'weak' PY tracts.

Enrichment of n-mers in the -80 to -30 region for introns with weak PY tracts versus all GC or AT-rich introns was determined (see Materials and methods). The entire list of enriched n-mers used in this study is available in Additional data files 2 and 3. According to this analysis, 99 n-mers were determined to be significantly enriched (*P *< 0.01) in the AT-rich class, and 349 n-mers were determined to be significantly enriched in the GC-rich class. For comparison, we drew random samples of the same size as the corresponding weak PY tract class for both the AT-rich and GC-rich introns, and determined enrichment using the same method as above. The average number of n-mers (for to seven nucleotides) that were determined to be significantly enriched in the randomly drawn samples was ten for the AT-rich and zero for the GC-rich class. Therefore, the enrichment measured appears to be strongly correlated with the composition of the PY tract as measured by the S_65 _score.

It has been proposed that signals that govern splicing of shorter (<200 nucleotides) introns may differ from those governing splicing of longer introns [51]. Therefore, we also evaluated short (<200 nucleotides) and long (≥ 200 nucleotides) AT-rich and GC-rich introns as independent classes. We found that enrichment was similar for both short and long GC-rich introns as evidenced by the observation that the enrichment score for n-mers correlated between these groups (Additional data file 6a). Meanwhile, little correlation was seen between the enrichment scores for long versus short AT-rich introns (Additional data file 6b). This is likely due to the fact that few n-mers were actually determined to be significantly enriched in the short AT-rich population (Additional data file 6b, and data not shown). Together, these data suggest that the compositional biases seen in the region upstream of the PY tract correlate with the potential for U2AF65 binding, especially for GC-rich introns, and that the bias is similar for both long and short introns.

**Figure 3:**
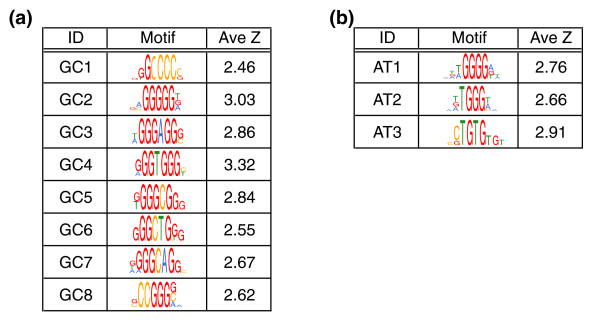
Introns containing weak PY tracts are enriched in specific motifs upstream of the PY tract. Shown are representative motifs derived from n-mers enriched in the region upstream of weak PY tracts (see Materials and methods for details of motif construction). The complete list of motifs is available in Additional data files 4 and 5. The average Z-score for enrichment of all of the n-mers that compose the motif is shown to the right. **(a) **Motifs over-represented upstream of weak PY tracts for GC-rich human introns. **(b) **Motifs over-represented upstream of weak PY tracts for AT-rich human introns.

To determine motifs, the enriched n-mers were clustered using the graph clustering method and software presented by Voelker and Berglund [52]. Clustering of the n-mers derived from the GC-rich introns yielded 25 clusters (Additional data file 4). These were manually separated into eight groups of compositionally similar motifs (Figure 3a). The n-mers derived from the AT-rich introns yielded eight clusters, of which the three most significant are shown in Figure 3b.

Motifs containing three to four contiguous guanidines are greatly enriched upstream of weak PY tracts for both AT-rich and GC-rich introns (Figure 3, motifs GC2-GC8 and AT1-AT2). Similar G-rich motifs have been previously shown to be enriched in this region [42,43]. G-rich intronic tracts have been shown to play important roles as splicing signals [53-56], and several heterogeneous nuclear ribonucleoproteins (hnRNPs), including hnRNPs A1, A2, F, and H, have been shown to bind G-rich RNA motifs [54,57-59]. The majority of the G-rich motifs appear to contain a common substring of three to four contiguous Gs separated by one to two nucleotides, and the preferred di-nucleotide spacers appear to be CT, CC, and CA.

In addition, we observed that C-rich motifs (containing three to four contiguous cytidines) are enriched upstream of weak GC-rich PY tracts (Figure 3, motif GC1). Using different computational methods, similar C-rich motifs have been predicted to be ISEs [60]. Our analysis provides additional evidence suggesting that C-rich motifs, located upstream of the PY tract, may play important roles in splicing.

We also observed that AT-rich introns with weak PY tracts were enriched in motifs similar to a motif recognized by the protein CUG-BP1 (Figure 3, motif AT3) [61]. It is interesting that these motifs did not appear in the GC-rich class. This may be due to compositional biases in the GC-rich class that preclude their identification using the computational methods that we employed, or it may imply that these motifs are, in fact, more abundantly represented in the AT-rich class.

These analyses demonstrate that certain motifs are statistically over-represented upstream of human introns containing weak PY tracts. We also wanted to assess how prevalent these motifs are among introns in general, and also determine the relative level of enrichment between introns with strong versus weak U2AF65 binding sites. Therefore, for each intron, we determined the percentage of the region from -80 to -30 that matched one or more of the n-mers determined to be enriched in introns with weak PY tracts relative to those with strong PY tracts (see above). We refer to this value as the percent coverage. As an example, 80% coverage indicates that 80% of the -80 to -30 region (or 40 of the 50 nucleotides) matches one or more of the enriched n-mers. This analysis (Additional data file 7) revealed that most introns have at least one match to an enriched n-mer. This is not surprising considering that the n-mers are only four to seven nucleotides in length, and, therefore, are expected to occur by chance with fairly high frequency. However, this analysis also revealed that introns with weak PY tracts are likely to have a greater coverage than introns with strong PY tracts. This is especially true of the GC-rich class of introns. For instance, while only 10% of GC-rich introns with strong PY tracts have 80-100% coverage, 23% of introns with weak PY tracts have this level of coverage (Additional data file 7). A smaller difference in coverage is seen between AT-rich introns with strong and weak PY tracts; however, the overall trend is the same (Additional data file 7). In both cases, the enriched n-mers tend to make up a greater portion of the -80 to -30 region for introns with weak PY tracts. Together, these observations indicate that the sequences represented by the enriched n-mers are rather common but they tend to cluster in introns with weak PY tracts.

### C-rich and G-rich motifs act as ISEs in an intron containing a weak polypyrimidine tract

LCAT intron 4 contains both C-rich and G-rich motifs upstream of the PY tract similar to those we identified computationally that are also highly conserved. The PY tract of LCAT intron 4 is a low-scoring PY tract and is not well conserved. To investigate the role of C-rich and G-rich motifs present in LCAT intron 4, we used a mini-gene system. We created a mini-gene that contains the last 50 nucleotides of LCAT intron 3, LCAT exon 4, LCAT intron 4, LCAT exon 5 and the first 50 nucleotides of LCAT intron 5. We included the downstream and upstream flanking introns in order to allow exon definition to occur, although short introns are often observed to function by intron definition [19].

### Mutation of the G-rich motifs

**Figure 4:**
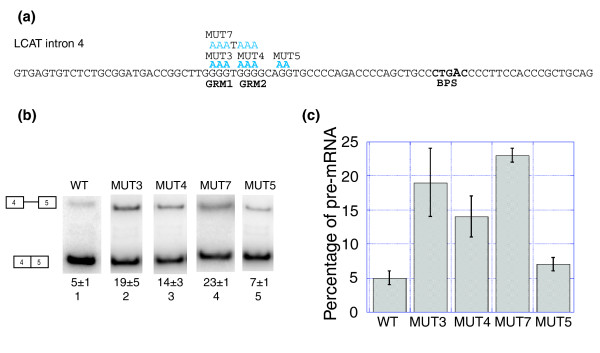
G-rich motifs function as ISEs in LCAT intron 4 splicing. **(a) **LCAT intron 4 with the mutations shown in blue above the WT sequence. BPS, branchpoint. **(b) **Splicing of the LCAT intron 4 mini-genes (WT, MUT3, MUT4, MUT7 and MUT 5) in HeLa cells. Splicing products (isolated from HeLa, reverse-transcribed and amplified with radioactive PCR) were resolved on an 8% non-denaturing gel and scanned using a phosphorimager. The pre-mRNA (top) is a 472 bp product and the mRNA (bottom) is a 389 bp product. The average quantification and standard deviation of the percent pre-mRNA (pre-mRNA divided by total RNA) for at least triplicate reactions is reported below each lane. **(c) **Graphical representation of the percent pre-mRNA for each LCAT mini-gene. Error bars represent standard deviation of replicate experiments.

We examined the role of two G-rich motifs (G-rich motif (GRM)1 and GRM2) present upstream of the PY tract of LCAT intron 4 (Figure 4a). The wild-type (WT) LCAT intron 4 mini-gene splices such that 5 ± 1% pre-mRNA is observed (Figure 4b, lane 1, and 4c). Mutation of GRM1 to AAA (MUT 3, Figure 4a) had a strong effect, and increased the unspliced product to 19 ± 5% (Figure 4b, lane 2, and 4c). Mutation of GRM2 to AAA (MUT 4, Figure 4a) had slightly less of an effect than MUT 3, resulting in 14 ± 3% pre-mRNA (Figure 4b, lane 3, and 4c). Mutation of both GRM1 and GRM2 (MUT 7, Figure 4a) had a similar effect as mutation of GRM1 alone (Figure 4b, lane 4, and 4c), suggesting that the two GRMs do not function additively towards recognition of LCAT intron 4. We also mutated a region that was neither a G-rich motif nor C-rich motif (MUT 5, Figure 4a) to be sure that the AAA motif we were inserting was not acting as an ISS. MUT 5 spliced similarly to WT (Figure 4b, compare lanes 1 and 5; Figure 4c), suggesting that the presence of the mutant AAA sequence in that region of LCAT intron 4 does not act as an ISS. These results suggest that GRM1 and GRM2 are ISEs important for the splicing of LCAT intron 4.

### Mutation of the C-rich motifs

**Figure 5:**
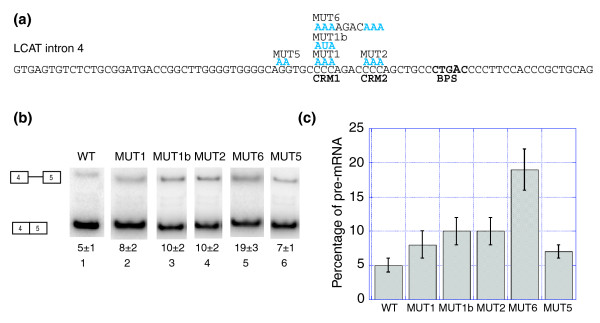
C-rich motifs function as ISEs in LCAT intron 4 splicing. **(a) **LCAT intron 4 with the mutations shown in blue above the WT sequence. BPS, branchpoint.** (b) **Splicing of the LCAT intron 4 mini-genes (WT, MUT1, MUT1b, MUT2, MUT 6 and MUT 5) in HeLa cells. Analysis was performed as in Figure 4. **(c) **Graphical representation of the percent pre-mRNA for each LCAT mini-gene. Error bars represent standard deviation of replicate experiments.

To determine whether the C-rich motifs function as ISEs, we mutated two C-rich motifs: C-rich motif (CRM)1 and CRM2 (Figure 5a), which are present upstream of the PY tract in LCAT intron 4. Mutation of CRM1 to AAA (MUT 1, Figure 5a) did not have a significant effect on splicing (Figure 5b, lane 2, and 5c). We also created a CRM1 mutant where we mutated CCC to AUA (MUT 1b, Figure 5a) and observed the same level of splicing as the AAA mutant (Figure 5b, compare lanes 2 and 3; Figure 5c). Similarly, mutation of CRM2 to AAA (MUT 2, Figure 5a) did not have a significant effect on splicing (Figure 5b, lane 4, and 5c). However, mutation of both CRM1 and CRM2 (MUT 6, Figure 5a) resulted in a decrease in splicing to 19 ± 3% pre-mRNA (Figure 5b, lane 5). These results suggest that while CRM1 and CRM2 do not individually contribute significantly to the splicing of LCAT intron 4, mutation of multiple C-rich motifs has a combinatorial effect.

### Cumulative mutation of the G-rich and C-rich motifs

**Figure 6:**
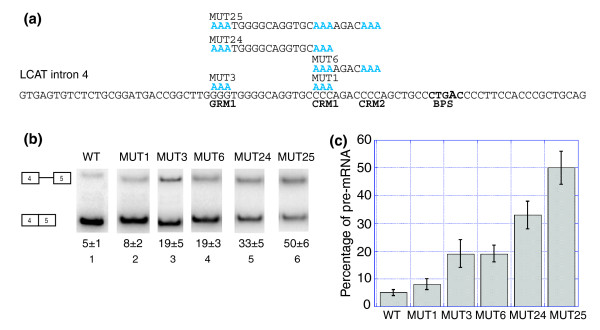
G-rich and C-rich motifs function combinatorially in LCAT intron 4 splicing. **(a) **LCAT intron 4 with the mutations shown in blue above the WT sequence. BPS, branchpoint. **(b) **Splicing of the LCAT intron 4 mini-genes (WT, MUT1, MUT3, MUT6, MUT 24 and MUT 25) in HeLa cells. Analysis was performed as in Figure 4. **(c) **Graphical representation of the percent pre-mRNA for each LCAT mini-gene. Error bars represent standard deviation of replicate experiments.

We hypothesized that the G-rich motifs and C-rich motifs could be functioning together in the recognition of LCAT intron 4. We have observed that there are many examples of introns where the G-rich and C-rich motifs are both present (data not shown). Mutation of both GRM1 and CRM1 (MUT 24, Figure 6a) resulted in a greater decrease in splicing (shown as an increase in percent pre-mRNA) than mutation of either motif alone (Figure 6b, compare MUT 24, lane 5, to MUT 1, lane 2, or MUT 3, lane 3; Figure 6c). An even greater decrease in splicing was observed for the combined mutation of GRM1, CRM1 and CRM2 (MUT 25, Figure 6b, compare MUT 25, lane 6, to MUT 3, lane 3 or MUT 6, lane 4; Figure 6c). These results suggest that the G-rich motifs and C-rich motifs function in combination to promote the splicing of LCAT intron 4.

### G-rich and C-rich motifs can functionally replace one another as ISEs

**Figure 7:**
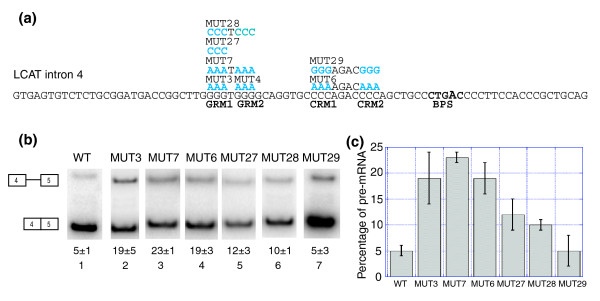
G-rich and C-rich motifs can functionally replace one another as ISEs. **(a) **LCAT intron 4 with the mutations shown in blue above the WT sequence. BPS, branchpoint.** (b) **Splicing of the LCAT intron 4 mini-genes (WT, MUT3, MUT7, MUT6, MUT 27, MUT 28 and MUT 29) in HeLa cells. Analysis was performed as in Figure 4. **(c) **Graphical representation of the percent pre-mRNA for each LCAT mini-gene. Error bars represent standard deviation of replicate experiments.

We examined whether the C-rich motifs could function in the place of the G-rich motifs. Mutation of GRM1 to CCC (MUT 27, Figure 7a) resulted in a smaller decrease in splicing compared to that observed for mutation of GRM1 to AAA (Figure 7b, compare MUT 27, lane 5, to MUT 3, lane 2; Figure 7c). Mutation of GRM1 and GRM2 to C-rich motifs (MUT 28, Figure 7a) also resulted in a smaller decrease in splicing compared to mutating GRM1 and GRM2 to AAA (Figure 7b, compare MUT 28, lane 6, to MUT 7, lane 3). We observed that both the single and double GRM to CRM mutations resulted in similar effects on splicing (Figure 7b, compare MUT 27, lane 5, to MUT 28, lane 6). These results suggest that a C-rich motif can partially compensate for a G-rich motif in this location. Furthermore, it appears that a C-rich motif followed by a G-rich motif (MUT 27) functions as effectively as two C-rich motifs (MUT 28). Mutation of CRM1 and CRM2 to G-rich motifs (MUT 29, Figure 7a) resulted in splicing similar to WT (Figure 7b, compare MUT 29, lane 7, to WT, lane 1; Figure 7c). We conclude that G-rich motifs can fully compensate for, and function in the place of, C-rich motifs, while C-rich motifs can only partially compensate for G-rich motifs.

### Strengthening the PY tract eliminates the role of the C-rich motifs

**Figure 8:**
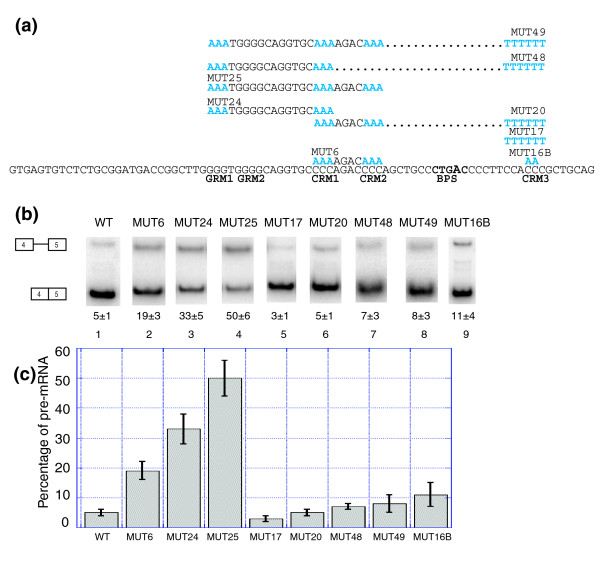
Strengthening the PY tract eliminates the role of the C-rich motifs. **(a) **LCAT intron 4 with the mutations shown in blue above the WT sequence. BPS, branchpoint.** (b) **Splicing of the LCAT intron 4 mini-genes (WT, MUT6, MUT24, MUT24, MUT17, MUT20, MUT48, MUT49 and MUT 16B) in HeLa cells. Analysis was performed as in Figure 4. **(c) **Graphical representation of the percent pre-mRNA for each LCAT mini-gene. Error bars represent standard deviation of replicate experiments.

We next investigated the role of the PY tract in LCAT intron 4 splicing. We mutated the PY tract to determine whether the C-rich sequences in the PY tract were also being recognized. Mutation of a C-rich sequence in the PY tract (CRM3, MUT 16B, Figure 8a) resulted in a minor decrease in splicing (MUT 16B, Figure 8b, lane 9, and 8c), indicating that CRM3 is not singly making a major contribution to the recognition of LCAT intron 4. However, the minor decrease in splicing does suggest that the PY tract may be playing a role. Strengthening the PY tract by mutating the sequence to include a run of eight uridines (MUT 17, Figure 8a) resulted in similar splicing to WT (Figure 8b, compare WT, lane 1, to MUT 17, lane 5). However, in the context of this strengthened PY tract, mutation of CRM1 and CRM2 (MUT 20, Figure 8a) did not result in decreased splicing (Figure 8b, compare MUT 20, lane 6, to MUT 6, lane 2; Figure 8c). Furthermore, the cumulative mutation of GRM1 and CRM1 (MUT 48, Figure 8a) or GRM1, CRM1 and CRM2 (MUT 49, Figure 8a) did not affect splicing in the presence of the strengthened PY tract (Figure 8b, compare MUT 48 to MUT 24 and MUT 49 to MUT 25). This result suggests that, in the context of a strengthened PY tract, the C-rich motifs and G-rich motifs are no longer necessary for recognition, while in the WT context the C-rich motifs and G-rich motifs function as ISEs to compensate for the weak LCAT intron 4 PY tract.

### C-rich motifs are ISEs in an additional intron containing a weak PY tract

**Figure 9:**
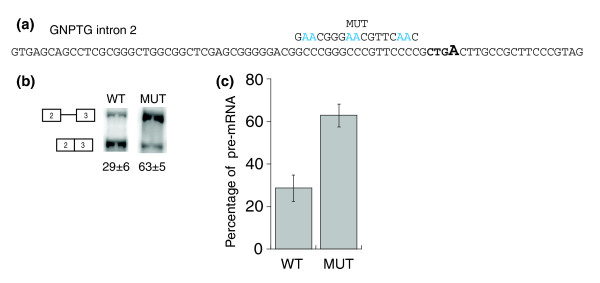
C-rich motifs function as ISEs in GNPTG intron 2. **(a) **GNPTG intron 2 with the mutations shown in blue above the WT sequence and the putative branchpoint sequence shown in bold.** (b) **Splicing of the GNPTG intron 2 mini-genes (WT and MUT) in HeLa cells. Splicing products (isolated from HeLa, reverse-transcribed and amplified with radioactive PCR) were resolved on a 10% non-denaturing gel and scanned using a phosphorimager. The average quantification and standard deviation of the percent pre-mRNA (pre-mRNA divided by total RNA) for triplicate reactions is reported below each lane. **(c) **Graphical representation of the percent pre-mRNA for the WT and MUT GNPTG mini-genes. Error bars represent standard deviation of replicate experiments.

GNPTG intron 2 is an alternatively spliced (intron retention) short intron containing multiple C-rich motifs upstream of a low scoring PY tract (Figure 9a, S_65 _score = 0.536). In order to test the function of the three C-rich motifs, we created a mini-gene containing exon 2, intron 2 and exon 3. The WT GNPTG intron 2 mini-gene splices such that 29 ± 6% pre-mRNA is observed (Figure 9b,c). Mutation of the three C-rich motifs upstream of the PY tract (Figure 9a) had a significant effect on splicing, resulting in 63 ± 5% pre-mRNA (Figure 9b,c). This result provides an additional example of C-rich motifs functioning as ISEs in an intron containing a weak PY tract.

## Discussion

The present model of pre-mRNA splicing is based on the recognition of the four canonical intronic motifs (5'ss, branchpoint sequence, PY tract and 3'ss) [3]. However, many introns lack one or more of these motifs and yet they are spliced. The diversity of human intronic sequences suggests that novel recognition pathways exist for non-canonical introns. Using an experimentally validated computational approach, introns lacking a canonical PY tract were isolated and analyzed to identify putative ISEs that functionally compensate in splicing when the PY tract is weak.

### U2AF65 binding to PY tracts confirms the U2AF65 SELEX scoring system

Our U2AF65 binding studies using various human intron PY tracts (Figure 2) confirm that the computational prediction can generally delineate strong and weak U2AF65 binding sites. Two caveats to our scoring system are: it is based solely on the U2AF65 SELEX data and, therefore, does not take into account nucleotide substitutions that are particularly deleterious for U2AF65 binding; and it cannot account for RNA secondary structure. Each of these parameters can contribute to lower than predicted binding affinities and may partially explain the deviations observed between predicted and observed binding strengths. Nevertheless, the S_65 _score is generally able to distinguish between sequences displaying strong and weak interactions with U2AF65, and it is more accurate than using simple uridine content alone.

For this analysis we also assume that the PY tract is located in the last 30 nucleotides of the intron. While this is a fair assumption for the vast majority of human introns, there are examples of introns where the PY tract and branchpoint sequence are located a further distance from the 3'ss AG [48,62-64]. Some of the human introns that score as having low scoring PY tracts may actually have high scoring PY tracts that are distally located. Although there are caveats to our scoring system, the S_65 _score generally distinguishes low and high affinity U2AF65 binding sites, allowing us to ask questions about the population of human introns with low affinity U2AF65 binding sites.

### Intronic motifs enriched upstream of weak PY tracts

We have identified families of motifs that are over-represented upstream of weak PY tracts but not upstream of strong PY tracts (Figure 3). Our evidence, combined with previous observations, suggests that these motifs function as ISEs that appear to compensate for weakened U2AF65-PY tract interactions. While we chose to focus our attention on the G-rich and C-rich triplet motifs, our study identified at least one additional motif that may represent binding sites for members of the CELF family of proteins. However, additional experimental evidence will need to be obtained to verify the functional significance of the other motifs identified by our study.

The experimental work presented here has focused on two relatively short introns, but our computational analysis found that the same families of motifs were over-represented in both short and long human introns (Additional data file 6). Although LCAT intron 4 is constitutively spliced, expressed sequence tag data suggest that GNPTG intron 2 is alternatively spliced, with some expressed sequence tags containing a retained intron 2. We expect to find examples where these motifs may play important roles in both constitutive and alternative splicing for both short and long introns.

### Interplay of G-rich and C-rich ISEs in the splicing of LCAT intron 4

G-rich motifs have been shown to be enriched in short mammalian introns [20,65]. The G-rich motif GRM1 is the strongest ISE we have observed in LCAT intron 4 (Figure 4). Double mutation of the two sequential G-rich motifs does not result in an additive effect on splicing. G-rich motifs have been shown to function in a combinatorial manner to promote splicing [20,56], although the spacing between G-rich motifs was greater (for example, 8-10 nucleotides [56]), than in LCAT intron 4, where only a single nucleotide separates the two G-rich motifs. Our studies confirm that G-rich sequences play an important role in promoting the recognition of GC-rich introns with weak PY tracts as previously observed [20].

Our results also show that C-rich motifs can act as ISEs like the G-rich motifs, but that the C-rich motifs may play more of an ancillary role to the G-rich motifs, at least in the case of LCAT intron 4 (Figure 5). C-rich motifs have been shown to function as an ISE in a chicken intron near the 5'ss [66], and as an ISS in a human intron near the 3'ss [67]. The single C-rich motif mutational studies presented here suggest that the C-rich motifs present in LCAT intron 4 have little individual effect on LCAT intron 4 splicing. However, we have observed that the C-rich motifs function additively, and that mutating both C-rich motifs (CRM1 and CRM2) is equivalent to mutating the one stronger G-rich motif (GRM1). Furthermore, we show that the mutation of multiple C-rich motifs in GNPTG intron 2 has a significant effect on splicing. This provides an additional example of C-rich motifs functioning as ISEs in an intron with a weak PY tract. C-rich motifs have not been previously shown to function as ISEs in human, nor to function in tandem to produce an additive effect on splicing.

Interestingly, the C-rich and G-rich motifs together function additively (Figure 6). This suggests a model where the combinatorial recognition of two separate ISEs promotes LCAT intron 4 splicing. It is intriguing to consider that interactions between the two ISEs could exist and represent an opportunity for protein-protein interactions between the C-rich and G-rich trans-factors, which could enhance intron recognition to a greater extent than either ISE (or trans-factor) alone. Combinatorial recognition of G-rich repeats with each other has been reported [20,55,56], as has the combinatorial function of a G-rich ISS with upstream ESSs [54]. Here we show that G-rich motifs can function in conjunction with C-rich ISEs to promote splicing, showing the flexibility of the G-rich motifs to function in different contexts.

We have also observed that the C-rich motifs can only partially compensate in the place of G-rich motifs in LCAT intron 4 splicing, while G-rich motifs appear to fully compensate in the place of C-rich motifs (Figure 7). It may be that the C-rich motifs have a positional dependence while the G-rich motifs do not. G-rich motifs appear to be the dominant ISE in LCAT intron 4 splicing. G-rich motifs appear to be capable of alleviating the need for C-rich motifs. The observation that C-rich motifs only partially rescue splicing reinforces the model that the C-rich motifs do not have the same enhancer strength as the G-rich motifs.

An examination of the primary sequence and predicted secondary structure (using mfold [68]) of LCAT intron 4 suggests that the intron could be folding into a stem-loop structure with the G-rich and C-rich sequences base-pairing (data not shown). While this is an intriguing model, when the C-rich motifs are replaced with G-rich motifs (a mutation that would abolish stem-loop formation; MUT 29, Figure 7), we observe splicing similar to WT levels, suggesting that a stem-loop structure is not contributing to the splicing of LCAT intron 4.

### Candidate protein factors for the G-rich and C-rich ISEs

There are multiple candidate proteins that could be recognizing the G-rich and C-rich motifs present in LCAT intron 4. A G-rich motif trans-factor, hnRNP H, has been identified and shown to bind G-rich sequences and regulate splicing both positively and negatively [54-56]. Several additional hnRNP proteins, including hnRNPs A1, A2, and F, have also been shown to bind G-rich RNA sequences [54,57-59]. An alternative model for G-rich sequence recognition involves RNA-RNA interactions to promote U1 snRNP binding. G-triplets near the 5'ss have been shown to bind the U1 snRNP by interacting with the U1 snRNA and this interaction was shown to be important for human alpha globin splicing *in vivo *[69].

hnRNP K and the α-CP proteins are the major poly-C-binding proteins identified in mammalian cells [70,71]. Both hnRNP K and several α-CP isoforms have been implicated in post-transcriptional control [70]. There have also been two studies that have implicated these proteins in splicing. hnRNP K was shown to enhance the splicing of a chicken b-Tropomyosin intron by binding a C-rich motif near the 5'ss [66]. A recent study has shown that α-CP2 binds a C-rich patch upstream of a weak PY tract in the human α-globin intron 1 transcript and inhibits splicing of this intron *in vitro *[67]. This is in contrast to our results with LCAT intron 4 where the C-rich motifs function as splicing enhancers, not silencers. Several *cis*-elements, including G-rich ISEs, have been shown to act as both splicing enhancers and silencers [54-56,72]. The C-rich motifs and their trans-factor may also possess the flexibility to function as silencers and enhancers.

### Role of the PY tract in splicing

**Figure 10:**
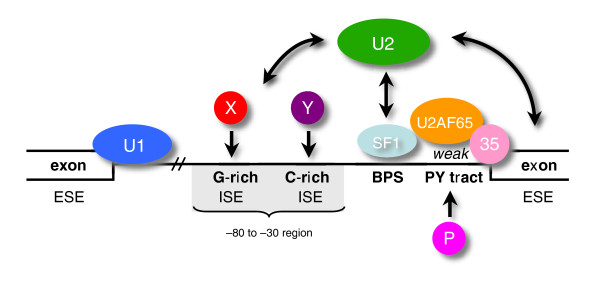
ISEs compensate for a weakened PY tract. The four factors present in the early (E) complex (U1 snRNP, SF1, U2AF65 and U2AF35) recognize the four canonical intronic splicing elements (the 5' splice site, the branchpoint (BPS), the PY tract and the 3' splice site). During A complex formation, which follows E complex, the U2 snRNP is recruited by U2AF65 and replaces SF1 at the branchpoint. There are presumably multiple redundant pathways that compensate for weak U2AF65-PY tract interactions, including bridging interactions between SF1, U2AF65 and U2AF35, alternative PY tract binding proteins (shown here as factor 'P'), and pathways involving additional non-canonical motifs such as ESEs or ISEs. We propose that ISEs in the region upstream of a weak PY tract (nucleotides -30 to -80) are important for recognizing introns with weak PY tracts. Specifically, we have shown that G-rich and C-rich motifs are ISEs that compensate for weakened U2AF65-PY tract interactions. Factors X and Y represent proteins binding the compensating ISEs. We propose that ISE-factor X/Y interactions can compensate for weak PY tract-U2AF65 interactions and help recruit the U2 snRNP to the branchpoint. The dash (//) indicates the variable length between the 5' splice site and 3' end of the intron.

Our results suggest a model where ISEs present upstream of a weak PY tract compensate for a weakened U2AF65-RNA interaction (Figure 10). In the case of LCAT intron 4, the G-rich and C-rich motifs and the branchpoint sequence are highly conserved and yet the PY tract is not well conserved. It is possible that the presence of strong enhancers upstream of the PY tract has allowed for greater degeneracy in the PY tract region. In support of this model we have observed that when the PY tract is strengthened to include a run of eight uridines, mutation of both C-rich motifs or the cumulative mutation of the G-rich and C-rich motifs no longer have an effect on LCAT intron 4 splicing (Figure 8). The G-rich and C-rich motifs appear dispensable in the presence of a strong PY tract. G-rich motifs have previously been shown to be dispensable for maximal splicing in the presence of a strengthened PY tract [20]. These results suggest that strong U2AF65-PY tract interactions alleviate the role of upstream ISEs.

An alternative model to weakened U2AF65-RNA interactions is that a splicing factor other than U2AF65 binds the weakened PY tracts. Recent work has shown that PUF60 plays a role in splicing by interacting with the PY tract [29]. Our observation that many of the weak PY tracts are particularly C-rich leads us to propose that a C-rich binding protein may function in this region. When we mutated a C-rich motif in the LCAT intron 4 PY tract we observed a small effect on splicing, suggesting that the C-rich motifs in the PY tract itself are recognized by a trans-factor (Figure 8). It is possible that such a splicing factor, be it U2AF65 or another protein, could be functioning in conjunction with the factor(s) that recognize the G-rich and C-rich ISEs upstream of the PY tract.

## Conclusion

### Novel mechanisms of intron recognition promote splicing of introns with non-canonical PY tracts

The pool of introns containing low-scoring U2AF65 binding sites represents a significant class of human introns lacking a canonical splicing element. The ISEs identified and validated here suggest that novel mechanisms exist in the cell for coping with weakened U2AF65-RNA interactions. Specifically, we have observed that the interplay of multiple *cis*-elements, in this case the G-rich and C-rich motifs, appears to be crucial for the recognition of non-canonical introns. In the future we plan to explore additional ISEs identified by this study to gain a broader picture of how the splicing machinery functions in the recognition of introns with weak PY tracts. While we have focused our attention here on a single key canonical splicing element, the PY tract, we plan on extending our analyses and expect to find that similar strategies exist for the recognition of other classes of non-canonical pre-mRNAs.

## Materials and methods

### Computational prediction of strength of U2AF65 binding sites in PY tracts

The March 2006 human reference sequence (NCBI Build 36.1) in conjunction with the UCSC KnownGenes (hg18) annotation database (Release 8 April 2007) [73] was used to create a non-redundant database of human intronic sequences. After excluding annotated introns that did not begin with G [T/C] [A/G] and end with AG, and were less than 60 bases in length, we were left with 171,475 unique acceptor ends.

In order to score PY tracts according to their likely affinity towards U2AF65, we developed a score that reflects the level of similarity of the PY tract sequence to the sequences that were enriched in RNAs derived from *in vitro *SELEX experiments using human U2AF65. In particular, if we let the frequency of occurrence of an n-mer *n *of length *k *within the SELEX sequences be represented by *f*_*n*_, then for a subject sequence of length *L *the S_65 _score is determined according to:

S65=∑i=0i=L−k+1ln⁡(fni14k)L−k+1

where *f*_*n *_represents the frequency (within the SELEX population) of the n-mer found at position *i *in the subject sequence.

The term ln⁡(fni14k) is a log-odds representation of the degree to which the particular n-mer was enriched within the SELEX sequences. Since the SELEX experiment began with uniformly random sequences, the denominator is simply the expectation for random occurrence of an n-mer of length *k*. For this study we chose the n-mer length to be five and the SELEX data were those reported in Singh *et al*. [27] and both SELEX experiments reported in Banerjee *et al*. [39]. The frequency of occurrence for all pentamers within these sequences is shown in Additional data file 1. Introns with 'strong' PY tracts (that is, expected to have high affinity for U2AF65) were defined to be those that are above the median value for all introns (0.811). All but one of the RNAs derived from *in vitro *SELEX had S_65 _scores above this value.

### Identification of intronic motifs over-represented upstream of weak PY tracts

In order to avoid biases due to long interspersed repetitive elements (LINEs) and short interspersed repetitive elements (SINEs), repetitive elements in the intronic sequence database (obtained as described above) were masked using the masking coordinates associated with the UCSC hg18 annotation database (Release 8 April 2007) [73]. However, simple repeats (many of which resemble known hnRNP binding sites) were not masked. The intronic acceptor sequences were then separated according to their GC content within the last 100 bases (or last half if the intron was less than 200 bases in length). AT-rich introns were defined to be introns containing less than 50% GC content. GC-rich introns were defined to be those containing greater than or equal to 50% GC content.

For each of these data sets, the occurrence of all n-mers (4-7 nucleotides) in the 50 nucleotide region from -80 to -30 (relative to the acceptor splice-junction) were determined using a sliding window. These counts were used to determine the background expectations for each n-mer. The occurrence of each 4-7 nucleotide n-mer within the equivalent region for all introns possessing 'weak' PY tracts (defined as above) was determined using a sliding window. From these values, n-mers that are enriched upstream of the branchpoint region for introns possessing weak PY tracts was determined using the binomial confidence interval method described in Voelker and Berglund [52]. For the AT-rich class, 99 n-mers were determined to be significantly enriched (*P *< 0.01), and 349 n-mers were determined to be significantly enriched for the GC-rich class. Enriched n-mers and corresponding counts and statistics are available in Additional data files 2 and 3. Enriched n-mers were used to construct motifs as in Voelker and Berglund [52]. All of the derived motifs and the identities and occurrences of all n-mers that were used to construct the motifs are available in Additional data files 4 and 5.

### U2AF65 binding

RNA oligonucleotides (listed in Figure 2b, IDT, Integrated DNA Technologies, San Diego, CA, USA) for U2AF65 binding assays were 5' end-labeled with γ-^32^P ATP using T4 polynucleotide kinase (NEB, Ipswich, MA, USA) for 30 minutes at 37°C. The RNAs were then gel purified using an 8% denaturing gel, eluted from the gel in 0.3M Na acetate and ethanol precipitated. The resulting pellet was resuspended in nanopure water and purified with a Bio-spin 6 column (BioRad, Hercules, CA, USA) equilibrated with nanopure water. The radioactivity level of the purified RNA solution was determined by scintillation. Gel-shift binding assays were performed using varying concentrations of recombinant human U2AF65 with constant amounts of radiolabeled RNA oligonucleotides as previously described [49]. The Ensembl gene accession numbers for the genes addressed in this study are: BRUNOL4 [ENSEMBL: ENSG00000101489], INSR [ENSEMBL: ENSG00000171105], LCAT [ENSEMBL: ENSG00000124067], MBNL1 [ENSEMBL: ENSG00000152601], SR140 [ENSEMBL: ENSG00000163714], and U2AF2 [ENSEMBL: ENSG00000063244].

### Cloning of mini-genes and mutants

WT LCAT intron 4 mini-gene was cloned from HeLa genomic DNA using primers to amplify the region between the last 50 nucleotides of LCAT intron 3 to the first 50 nucleotides of LCAT intron 5 (502 nucleotides). The forward primer included a *Bam*H1 site and the reverse primer included an *Eco*R1 site. The amplified genomic DNA was cut with *Bam*H1 and *Eco*R1, inserted into pcDNA3 and sequenced. LCAT intron 4 mutants were made by PCR using the WT LCAT 4 mini-gene as template and primers containing the mutation of interest. LCAT intron 4 mutants were also cloned into pcDNA3 using *Bam*H1 and *Eco*R1 and sequenced. The WT and mutant GNPTG [ENSEMBL: ENSG00000090581] intron 2 mini-genes were cloned using overlapping primers to create a sequence containing exon 2, intron 2 and exon 3. This sequence was flanked by cut sites *Hin*dIII and *Not*1, cloned into pcDNA3 and sequenced.

### *In vivo *splicing assays: cell culture, transfection, and harvesting

HeLa cells were grown in monolayers in DMEM with GLUTAMAX (Invitrogen, Carlsbad, CA, USA) and supplemented with 10% fetal bovine serum (GIBCO). For the LCAT splicing experiments 1.5 (± 0.2) × 10^5 ^cells were plated in 6-well plates and transfected 18-20 h later at approximately 70% confluency. Plasmid (1 μg) was transfected into each well of cells using 5 μl of Lipofectin (Invitrogen, Carlsbad, CA, USA) and 10 μl of Plus reagent (Invitrogen) according to the manufacturer's protocols. For the GNPTG splicing experiments, 2 × 10^5 ^cells were plated in 6-well plates and transfected with 1 μg plasmid 18-20 h later using 5 μl of Lipofectamine 2000. Cells were harvested 24 h (LCAT experiments) or 16 h (GNPTG experiments) after transfection using TriplE (GIBCO) and then pelleted by centrifugation. RNA was isolated from the cell pellets using an RNeasy kit (QIAGEN, Valencia, CA, USA).

### *In vivo *splicing assays: DNAsing, reverse transcription, PCR, and quantifying percent mRNA

Isolated RNA (500 ng) was incubated with 1 unit of RQI DNase (Promega, Madison, WI, USA) in a 10 μl reaction for 2 h (LCAT experiments) or 1 h (GNPTG experiments) according to the manufacturer's protocol. DNAsed RNA (2 μl (100 ng)) was reverse transcribed in a 10 μl reaction (1:5 dilution) using Superscript II and an LCAT-specific reverse primer or a reverse primer to the pCDNA3 SP6 sequence for the GNPTG experiments, according to manufacturer's protocols with the exception that we used half the recommended amount of Superscript II (Invitrogen, Carlsbad, CA, USA). For the LCAT splicing experiments, 2 μl of the reverse transcription reaction was subjected to 20 rounds of PCR amplification in a 20 μl reaction (1:10 dilution) using LCAT specific primers spiked with a kinased LCAT forward primer (0.4 nM). Twenty rounds of PCR were found to be within the linear range for this PCR experiment (data not shown). The resulting PCR products were run on an 8% (19:1) polyacrylamide native gel. For the GNPTG splicing experiments, 2 μl of the reverse transcription reaction was subjected to 27 rounds of PCR amplification in a 20 μl reaction (1:10 dilution) using primers specific to the T7 (forward) and SP6 (reverse) sequences of the pcDNA3 plasmid spiked with kinased T7 forward primer. Twenty-seven rounds of PCR were found to be within the linear range for this PCR experiment (data not shown). The resulting PCR products were run on a 10% (19:1) polyacrylamide gel. The gels were dried and exposed overnight to a phosphorimager screen. Quantification of the radioactive bands was performed using ImageQuant software (GE Healthcare, London, UK). The percent pre-mRNA was calculated by dividing the amount of the pre-mRNA band by the total amount of the pre-mRNA and mRNA bands and multiplying by 100%.

## Abbreviations

ADML, adenovirus major late; CRM, C-rich motif; ESE, exonic splicing enhancer; ESS, exonic splicing silencer; GNPTG, N-acetylglucosamine-1-phosphotransferase gamma subunit; GRM, G-rich motif; hnRNP, heterogeneous nuclear ribonucleoproteins; ISE, intronic splicing enhancer; ISS, intronic splicing silencer; LCAT, lecithin cholesterol acyltransferase; PY tract, polypyrimidine tract; S_65 _score, U2AF65 binding site score; SF, splicing factor; snRNP, small nuclear ribonucleoprotein; ss, splice site; U2AF, U2 snRNP auxilliary factor; WT, wild-type.

## Authors' contributions

JIM and RBV designed experiments, performed experiments, analyzed data and wrote the paper. KLH and MBW performed experiments and analyzed data. JAB designed experiments, analyzed data and wrote the paper.

## Additional data files

The following additional data files are available with the online version of this paper. Additional data file 1 is a table listing the probability of occurrence for pentamers found in U2AF65 SELEX derived sequences. Additional data file 2 is a table listing the n-mers enriched upstream of weak PY tracts from GC-rich introns. Additional data file 3 is a table listing the n-mers enriched upstream of weak PY tracts from AT-rich introns. Additional data file 4 is a table listing the clusters enriched upstream of weak PY tracts from GC-rich introns. Additional data file 5 is a table listing the clusters enriched upstream of weak PY tracts from AT-rich introns. Additional data file 6 is a figure of the scatterplots of Z-scores for enrichment upstream of weak PY tracts for long versus short introns. Additional data file 7 is a histogram showing the percentage of introns possessing specific n-mers that are enriched upstream of weak PY tracts

## Supplementary Material

Additional data file 1 Table listing the count and the probability of occurrence (using a sliding window) for all pentamers found in the sequences reported in Singh *et al*. [27] and both SELEX experiments reported in Banerjee *et al*. [39]. Click here for file

Additional data file 2 Associated statistics and listing of n-mers (4-7 nucleotides) determined to be enriched in the 50 nucleotide region upstream of weak PY tracts from GC-rich introns. Click here for file

Additional data file 3 Associated statistics and listing of n-mers (4-7 nucleotides) determined to be enriched in the 50 nucleotide region upstream of weak PY tracts from AT-rich introns. Click here for file

Additional data file 4 Listing of all clusters derived from n-mers enriched in the 50 nucleotide region upstream of weak PY tracts from GC-rich introns. Included are the individual n-mers and associated statistics used to produce each motif. Click here for file

Additional data file 5 Listing of all clusters derived from n-mers enriched in the 50 nucleotide region upstream of weak PY tracts from AT-rich introns. Included are the individual n-mers and associated statistics used to produce each motif. Click here for file

Additional data file 6 The Z-scores for enrichment of all 4-7 nucleotide n-mers in the intronic region upstream (-80 to -30 relative to the acceptor splice-junction) of PY tracts with low S_65 _scores for short (<200 nucleotide) introns is plotted versus long (≥ 200 nucleotide) introns. **(a) **Data for GC-rich introns. **(b) **Data for AT-rich introns. Click here for file

Additional data file 7 The portion of the sequence corresponding to the -80 to -30 region matching one or more of the n-mers enriched in the same region for introns with weak PY tracts (Additional data files 2 and 3) was determined. These values (referred to as the percent coverage) were binned as indicated along the x-axis. Click here for file
