# Supplementary material for: Identification of motifs that function in the splicing of non-canonical introns
Source: Genome Biol. 2008 Jun 12;9(6):R97. doi: 10.1186/gb-2008-9-6-r97 (PMC2481429; doi:10.1186/gb-2008-9-6-r97)
Supplement: Additional data file 1 — Table listing the count and the probability of occurrence (using a sliding window) for all pentamers found in the sequences reported in Singh et al. [27] and both SELEX experiments reported in Banerjee et al. [39]. [file gb-2008-9-6-r97-S1.pdf]

N-mer	Count	Prob
TTTTT	110	0.053114
TTTTC	72	0.034766
TTTCC	51	0.024626
TTTCT	48	0.023177
TCTTT	43	0.020763
CTTTT	42	0.020280
TTCTT	42	0.020280
GTTTT	41	0.019797
TTCCC	38	0.018349
TGTTT	28	0.013520
TTCTC	26	0.012554
CTTTC	21	0.010140
TCCTT	20	0.009657
TTTTG	20	0.009657
TTCCT	19	0.009174
TTTGT	19	0.009174
TCTCC	18	0.008691
TCCCT	17	0.008209
TTGTT	17	0.008209
TCCCC	16	0.007726
CCTTT	15	0.007243
ATTTT	13	0.006277
CATTT	13	0.006277
CCTTC	13	0.006277
CGTTT	13	0.006277
CTCTT	13	0.006277
CTTCT	13	0.006277
TCTTC	13	0.006277
GTTCT	12	0.005794
TCTCT	12	0.005794
CCCTT	11	0.005311
CTTCC	11	0.005311
AGTTT	10	0.004829
CCATT	10	0.004829
TCCAT	10	0.004829
TTATT	10	0.004829
TTCCA	10	0.004829
TTCGT	10	0.004829
ACTTT	9	0.004346
ATTTC	9	0.004346
GTTTC	9	0.004346
TCCCA	9	0.004346
TCGTT	9	0.004346
TGTCC	9	0.004346
TGTTC	9	0.004346
TTTCG	9	0.004346
CCTGT	8	0.003863
CTCCC	8	0.003863
CTCCT	8	0.003863
CTCTC	8	0.003863
CTGTT	8	0.003863
TTGTC	8	0.003863
ACGTT	7	0.003380
ACTCT	7	0.003380
CACTT	7	0.003380
CGTTC	7	0.003380
CTTAT	7	0.003380

N-mer	Count	Prob
TCATT	7	0.003380
TCCTG	7	0.003380
TGATT	7	0.003380
TTTAT	7	0.003380
ACCTT	6	0.002897
ATGTT	6	0.002897
ATTCC	6	0.002897
CCTTA	6	0.002897
CTTCG	6	0.002897
GTCCC	6	0.002897
GTGTT	6	0.002897
TATTT	6	0.002897
TCCAC	6	0.002897
TCTGT	6	0.002897
TTCAT	6	0.002897
TTTGG	6	0.002897
AATTC	5	0.002414
ATACT	5	0.002414
ATCTT	5	0.002414
ATTCT	5	0.002414
CAGTT	5	0.002414
CCACT	5	0.002414
CCCTG	5	0.002414
CTCCA	5	0.002414
CTGAT	5	0.002414
CTTGT	5	0.002414
CTTTA	5	0.002414
GATTT	5	0.002414
GCCTC	5	0.002414
GCTTT	5	0.002414
GTCCA	5	0.002414
GTCCT	5	0.002414
TACGT	5	0.002414
TACTC	5	0.002414
TATTC	5	0.002414
TCCTA	5	0.002414
TTACT	5	0.002414
TTATC	5	0.002414
TTCTG	5	0.002414
TTGAT	5	0.002414
TTTAC	5	0.002414
TTTCA	5	0.002414
TTTGC	5	0.002414
AATTT	4	0.001931
ATTTG	4	0.001931
CACCT	4	0.001931
CATGT	4	0.001931
CATTC	4	0.001931
CATTG	4	0.001931
CCACC	4	0.001931
CCCCT	4	0.001931
CCCTC	4	0.001931
CCTCT	4	0.001931
CCTGA	4	0.001931
CCTTG	4	0.001931
CGACC	4	0.001931
CGCCT	4	0.001931

N-mer	Count	Prob
CTACT	4	0.001931
CTGTC	4	0.001931
CTTTG	4	0.001931
GGTTT	4	0.001931
GTTTA	4	0.001931
GTTTG	4	0.001931
TAACC	4	0.001931
TACAT	4	0.001931
TACTT	4	0.001931
TAGTT	4	0.001931
TATCT	4	0.001931
TATTA	4	0.001931
TCCCG	4	0.001931
TCGTC	4	0.001931
TCTTA	4	0.001931
TGCTT	4	0.001931
TGTGT	4	0.001931
TTATG	4	0.001931
TTCTA	4	0.001931
TTGCC	4	0.001931
TTTTA	4	0.001931
AAGTT	3	0.001449
AATCT	3	0.001449
ACATA	3	0.001449
ACATT	3	0.001449
AGTCC	3	0.001449
AGTGT	3	0.001449
AGTTA	3	0.001449
ATATT	3	0.001449
ATCCC	3	0.001449
ATTAC	3	0.001449
ATTCA	3	0.001449
ATTGA	3	0.001449
ATTGT	3	0.001449
ATTTA	3	0.001449
CAATT	3	0.001449
CACAT	3	0.001449
CACTC	3	0.001449
CATAC	3	0.001449
CCAAT	3	0.001449
CCAGT	3	0.001449
CCATG	3	0.001449
CCCAC	3	0.001449
CCCAT	3	0.001449
CCCGT	3	0.001449
CCGTT	3	0.001449
CCTAA	3	0.001449
CCTCC	3	0.001449
CCTCG	3	0.001449
CGTCC	3	0.001449
CTAAC	3	0.001449
CTACG	3	0.001449
CTCTG	3	0.001449
CTTAC	3	0.001449
GAGCT	3	0.001449
GATTC	3	0.001449
GCTAC	3	0.001449

N-mer	Count	Prob
GTCGT	3	0.001449
GTCTT	3	0.001449
GTTAT	3	0.001449
GTTCC	3	0.001449
TAATC	3	0.001449
TCACT	3	0.001449
TCCAA	3	0.001449
TCGAT	3	0.001449
TCGCT	3	0.001449
TCTAC	3	0.001449
TCTCA	3	0.001449
TCTCG	3	0.001449
TCTTG	3	0.001449
TGATA	3	0.001449
TGCCT	3	0.001449
TGTCT	3	0.001449
TTAAT	3	0.001449
TTACA	3	0.001449
TTACC	3	0.001449
TTAGT	3	0.001449
TTCAC	3	0.001449
TTCGA	3	0.001449
TTGCT	3	0.001449
TTGGT	3	0.001449
TTGTG	3	0.001449
TTTAG	3	0.001449
TTTGA	3	0.001449
AAAGT	2	0.000966
AATAC	2	0.000966
AATCC	2	0.000966
ACACT	2	0.000966
ACCAT	2	0.000966
ACGGA	2	0.000966
ACTAC	2	0.000966
ACTCC	2	0.000966
ACTGT	2	0.000966
AGCTA	2	0.000966
AGCTT	2	0.000966
ATACA	2	0.000966
ATACG	2	0.000966
ATCGA	2	0.000966
ATCTC	2	0.000966
ATTAA	2	0.000966
ATTAT	2	0.000966
ATTGC	2	0.000966
CAAAG	2	0.000966
CAATA	2	0.000966
CACCG	2	0.000966
CACGT	2	0.000966
CAGTC	2	0.000966
CATAT	2	0.000966
CATCG	2	0.000966
CATTA	2	0.000966
CCAAC	2	0.000966
CCACG	2	0.000966
CCATA	2	0.000966
CCATC	2	0.000966

N-mer	Count	Prob
CCCAA	2	0.000966
CCCAG	2	0.000966
CCCGA	2	0.000966
CCCTA	2	0.000966
CCGAC	2	0.000966
CCGGT	2	0.000966
CCGTA	2	0.000966
CCTAG	2	0.000966
CCTAT	2	0.000966
CCTGC	2	0.000966
CCTGG	2	0.000966
CGAGC	2	0.000966
CGATG	2	0.000966
CGCTA	2	0.000966
CGCTT	2	0.000966
CGGAT	2	0.000966
CGGTT	2	0.000966
CGTAA	2	0.000966
CTAGT	2	0.000966
CTATA	2	0.000966
CTATT	2	0.000966
CTCAT	2	0.000966
CTCGA	2	0.000966
CTCGC	2	0.000966
CTGTG	2	0.000966
CTTAG	2	0.000966
CTTGC	2	0.000966
GAATT	2	0.000966
GACCT	2	0.000966
GAGTT	2	0.000966
GATAC	2	0.000966
GATAT	2	0.000966
GATCG	2	0.000966
GATTG	2	0.000966
GCATT	2	0.000966
GCCGT	2	0.000966
GCCTG	2	0.000966
GCGCC	2	0.000966
GCTCC	2	0.000966
GCTTA	2	0.000966
GCTTC	2	0.000966
GTGTC	2	0.000966
GTTCA	2	0.000966
TAATT	2	0.000966
TACAC	2	0.000966
TACCA	2	0.000966
TACTA	2	0.000966
TACTG	2	0.000966
TAGCT	2	0.000966
TATAC	2	0.000966
TATCC	2	0.000966
TATGC	2	0.000966
TATGT	2	0.000966
TATTG	2	0.000966
TCAAT	2	0.000966
TCAGT	2	0.000966
TCGAC	2	0.000966

N-mer	Count	Prob
TCGAG	2	0.000966
TGAGT	2	0.000966
TGGCC	2	0.000966
TGGTT	2	0.000966
TGTCG	2	0.000966
TGTTG	2	0.000966
TTAGC	2	0.000966
TTCCG	2	0.000966
TTCGC	2	0.000966
TTTAA	2	0.000966
AAAAC	1	0.000483
AAACT	1	0.000483
AAAGC	1	0.000483
AAATT	1	0.000483
AACAC	1	0.000483
AACCA	1	0.000483
AACCG	1	0.000483
AACCT	1	0.000483
AACGG	1	0.000483
AACTA	1	0.000483
AACTC	1	0.000483
AAGAT	1	0.000483
AAGCA	1	0.000483
AAGCC	1	0.000483
AATGT	1	0.000483
AATTA	1	0.000483
ACAAA	1	0.000483
ACACC	1	0.000483
ACATC	1	0.000483
ACCAG	1	0.000483
ACCCC	1	0.000483
ACCGA	1	0.000483
ACCGG	1	0.000483
ACCTA	1	0.000483
ACCTG	1	0.000483
ACGAC	1	0.000483
ACGGC	1	0.000483
ACTAA	1	0.000483
ACTAT	1	0.000483
ACTCA	1	0.000483
ACTGA	1	0.000483
ACTTA	1	0.000483
ACTTC	1	0.000483
AGATA	1	0.000483
AGATT	1	0.000483
AGCAC	1	0.000483
AGCCC	1	0.000483
AGCCG	1	0.000483
AGCCT	1	0.000483
AGCGC	1	0.000483
AGCTC	1	0.000483
AGTGC	1	0.000483
AGTTC	1	0.000483
ATAAG	1	0.000483
ATATA	1	0.000483
ATATC	1	0.000483
ATCAT	1	0.000483

N-mer	Count	Prob
ATCCA	1	0.000483
ATCCT	1	0.000483
ATCGC	1	0.000483
ATCGG	1	0.000483
ATCGT	1	0.000483
ATCTG	1	0.000483
ATGAG	1	0.000483
ATGAT	1	0.000483
ATGCA	1	0.000483
ATGCG	1	0.000483
ATGCT	1	0.000483
ATGGA	1	0.000483
ATGTC	1	0.000483
ATTCG	1	0.000483
CAAAA	1	0.000483
CAACA	1	0.000483
CAACC	1	0.000483
CAACT	1	0.000483
CAATC	1	0.000483
CAATG	1	0.000483
CACGG	1	0.000483
CAGAT	1	0.000483
CAGCC	1	0.000483
CAGTG	1	0.000483
CATCA	1	0.000483
CATCC	1	0.000483
CATCT	1	0.000483
CCAAA	1	0.000483
CCAGA	1	0.000483
CCAGC	1	0.000483
CCCCA	1	0.000483
CCCCC	1	0.000483
CCCCG	1	0.000483
CCCGG	1	0.000483
CCGAG	1	0.000483
CCGAT	1	0.000483
CCGCC	1	0.000483
CCGGA	1	0.000483
CCGGG	1	0.000483
CCGTC	1	0.000483
CCTAC	1	0.000483
CCTCA	1	0.000483
CGAAT	1	0.000483
CGACG	1	0.000483
CGAGT	1	0.000483
CGATT	1	0.000483
CGCAA	1	0.000483
CGCAG	1	0.000483
CGCAT	1	0.000483
CGCCG	1	0.000483
CGGAA	1	0.000483
CGGCC	1	0.000483
CGGCT	1	0.000483
CGGGT	1	0.000483
CGTAC	1	0.000483
CGTAG	1	0.000483
CGTCG	1	0.000483

N-mer	Count	Prob
CGTCT	1	0.000483
CGTGT	1	0.000483
CTAAT	1	0.000483
CTACA	1	0.000483
CTACC	1	0.000483
CTAGC	1	0.000483
CTATC	1	0.000483
CTATG	1	0.000483
CTCAA	1	0.000483
CTCAC	1	0.000483
CTCCG	1	0.000483
CTCGT	1	0.000483
CTGCC	1	0.000483
CTGCT	1	0.000483
CTGGA	1	0.000483
CTGGC	1	0.000483
CTGGT	1	0.000483
CTGTA	1	0.000483
CTTAA	1	0.000483
GAAAG	1	0.000483
GACCA	1	0.000483
GACCG	1	0.000483
GACGA	1	0.000483
GACGG	1	0.000483
GACTC	1	0.000483
GAGTG	1	0.000483
GATGC	1	0.000483
GATGG	1	0.000483
GCAAA	1	0.000483
GCAAC	1	0.000483
GCAAT	1	0.000483
GCACC	1	0.000483
GCAGT	1	0.000483
GCATC	1	0.000483
GCCAA	1	0.000483
GCCCA	1	0.000483
GCCCG	1	0.000483
GCCCT	1	0.000483
GCCGG	1	0.000483
GCCTA	1	0.000483
GCCTT	1	0.000483
GCGCT	1	0.000483
GCGGA	1	0.000483
GCGTA	1	0.000483
GCTAT	1	0.000483
GCTCT	1	0.000483
GGAAA	1	0.000483
GGAAT	1	0.000483
GGACG	1	0.000483
GGACT	1	0.000483
GGATC	1	0.000483
GGATG	1	0.000483
GGCAT	1	0.000483
GGCCA	1	0.000483
GGCCC	1	0.000483
GGCTC	1	0.000483
GGCTT	1	0.000483

N-mer	Count	Prob
GGGAC	1	0.000483
GGGCA	1	0.000483
GGGTA	1	0.000483
GGTCC	1	0.000483
GTAAC	1	0.000483
GTAAG	1	0.000483
GTAAT	1	0.000483
GTACA	1	0.000483
GTAGT	1	0.000483
GTATT	1	0.000483
GTCTA	1	0.000483
GTGAG	1	0.000483
GTGCC	1	0.000483
GTGGC	1	0.000483
GTGTA	1	0.000483
GTTGA	1	0.000483
GTTGC	1	0.000483
TAAAT	1	0.000483
TAACG	1	0.000483
TAAGA	1	0.000483
TAAGC	1	0.000483
TACAA	1	0.000483
TACCC	1	0.000483
TACCT	1	0.000483
TAGCC	1	0.000483
TAGTC	1	0.000483
TAGTG	1	0.000483
TATAA	1	0.000483
TATCG	1	0.000483
TATGA	1	0.000483
TCACA	1	0.000483
TCATC	1	0.000483
TCATG	1	0.000483
TCCAG	1	0.000483
TCCGA	1	0.000483
TCCGC	1	0.000483
TCCGG	1	0.000483
TCCGT	1	0.000483
TCCTC	1	0.000483
TCGCA	1	0.000483
TCGCC	1	0.000483
TCGGC	1	0.000483
TCGTA	1	0.000483
TCGTG	1	0.000483
TCTAG	1	0.000483
TCTAT	1	0.000483
TCTGC	1	0.000483
TGAGC	1	0.000483
TGATC	1	0.000483
TGCAA	1	0.000483
TGCAT	1	0.000483
TGCCC	1	0.000483
TGCCG	1	0.000483
TGCGC	1	0.000483
TGCGG	1	0.000483
TGCGT	1	0.000483
TGCTC	1	0.000483

N-mer	Count	Prob
TGGAA	1	0.000483
TGGAC	1	0.000483
TGGAT	1	0.000483
TGGCT	1	0.000483
TGGGA	1	0.000483
TGGTC	1	0.000483
TGTAA	1	0.000483
TGTAT	1	0.000483
TGTGA	1	0.000483
TGTGG	1	0.000483
TTAAA	1	0.000483
TTAAC	1	0.000483
TTCAA	1	0.000483
TTCAG	1	0.000483
TTGAG	1	0.000483
TTGCA	1	0.000483
TTGCG	1	0.000483
TTGGA	1	0.000483
TTGGC	1	0.000483
TTGGG	1	0.000483
