# Supplementary material for: Identification of motifs that function in the splicing of non-canonical introns
Source: Genome Biol. 2008 Jun 12;9(6):R97. doi: 10.1186/gb-2008-9-6-r97 (PMC2481429; doi:10.1186/gb-2008-9-6-r97)
Supplement: Additional data file 2 — Associated statistics and listing of n-mers (4-7 nucleotides) determined to be enriched in the 50 nucleotide region upstream of weak PY tracts from GC-rich introns. [file gb-2008-9-6-r97-S2.pdf]

Nmers enriched upstream of weak PY tracts for GC-rich introns

**Field**	**Description**
Nmer	The Nmer evaluated
Count(S)	The observed counts for the nmer in the sample
Count(P)	The observed counts for the nmer in the background
Prob(S)	Probability of occurrence for the nmer in the sample
Prob(P)	Probability of occurrence for the nmer in the background
CI(low)	Lower confidence interval (P<0.01) based upon binomial distribution
CI(high)	Upper confidence interval (P<0.01) based upon binomial distribution
Z_score	Z score (based upon binomial distribution)
P(Z)	Probability of observation
E(S)	Expected occurrence in sample (based upon sample size and background probabilities)
E(CI_low)	Minimal occurrence in sample expected by chance (based upon sample size and lower CI)
E(CI_high)	Maximum occurrence in sample expected by chance (based upon sample size and upper CI)

Nmer	Count(S)	Count(P)	Prob(S)	Prob(P)	CI(low)	CI(high)	Z_score	P(Z)	E(S)	E(CI_low)	E(CI_high)
GGGG	35801	46210	0.0176	0.0163	0.0161	0.0165	11.43	2.81E-30	33054.8	32638	33477
GGGC	29216	38184	0.0560	0.0524	0.0517	0.0531	8.82	1.14E-18	27331.6	26959.6	27708.4
TGGG	34125	45037	0.0654	0.0618	0.0610	0.0626	8.20	2.49E-16	32236.9	31834.7	32643.8
CGGG	10624	13383	0.0204	0.0184	0.0179	0.0188	8.05	8.51E-16	9579.37	9356.37	9807.58
GGGT	20020	26265	0.0384	0.0360	0.0354	0.0366	6.83	8.52E-12	18800.1	18489.5	19115.8
AGGG	26367	35015	0.0505	0.0480	0.0474	0.0487	6.38	1.79E-10	25063.3	24706.4	25425.1
GGTG	21503	28401	0.0310	0.0293	0.0288	0.0298	6.34	2.30E-10	20323	19998.8	20652.3
TGGGG	13314	17326	0.0308	0.0287	0.0281	0.0293	6.31	2.72E-10	12393.2	12140.5	12651
GGGGG	8965	11478	0.0045	0.0041	0.0040	0.0042	6.28	3.35E-10	8207.15	7999.5	8420.17
GGGGC	10751	13880	0.0249	0.0230	0.0225	0.0235	6.27	3.54E-10	9928.29	9701.74	10160
GTGGG	10631	13730	0.0205	0.0189	0.0185	0.0194	6.19	6.13E-10	9822.27	9596.49	10053.2
GGGTG	9729	12521	0.0187	0.0173	0.0168	0.0177	6.17	6.76E-10	8957.36	8741.69	9178.26
GCGG	7708	9827	0.0111	0.0101	0.0099	0.0104	6.07	1.30E-09	7031.95	6840.49	7228.7
GTGG	22703	30113	0.0327	0.0311	0.0306	0.0315	6.04	1.57E-09	21548.1	21214.5	21886.8
GGCG	7489	9551	0.0108	0.0098	0.0096	0.0101	5.96	2.55E-09	6834.45	6645.71	7028.49
GAGG	23734	31561	0.0342	0.0325	0.0321	0.0330	5.88	4.14E-09	22584.2	22242.9	22930.6
GGCC	24994	33288	0.0479	0.0457	0.0450	0.0463	5.85	4.88E-09	23827.1	23478.8	24180.4
GGGGT	7591	9715	0.0176	0.0161	0.0156	0.0165	5.82	5.95E-09	6949.09	6759.33	7144.08
GGAG	23973	31923	0.0345	0.0329	0.0324	0.0334	5.75	9.13E-09	22843.3	22500	23191.6
GGGA	22880	30438	0.0439	0.0418	0.0411	0.0424	5.72	1.08E-08	21787.1	21453.4	22125.7
GCGGG	3687	4554	0.0071	0.0063	0.0060	0.0065	5.61	2.05E-08	3257.87	3128.15	3392.94

CGGGG	4255	5311	0.0099	0.0088	0.0085	0.0091	5.54	3.04E-08	3798.93	3658.81	3944.37
GGTGG	9986	12988	0.0145	0.0134	0.0131	0.0138	5.48	4.37E-08	9289.03	9068.95	9514.38
GGTGGG	5025	6355	0.0097	0.0088	0.0085	0.0091	5.36	8.34E-08	4543.77	4390.28	4702.57
GGGAG	10466	13658	0.0202	0.0188	0.0184	0.0193	5.34	9.08E-08	9770.76	9545.57	10001.2
GGGTGG	4768	6038	0.0092	0.0084	0.0081	0.0087	5.15	2.58E-07	4317.11	4167.54	4472.01
GGAGG	10852	14226	0.0157	0.0147	0.0144	0.0151	5.10	3.43E-07	10174.4	9944.14	10410
CCGG	7945	10301	0.0152	0.0141	0.0138	0.0145	5.04	4.67E-07	7373.32	7177.59	7574.3
GGGCG	3407	4244	0.0066	0.0058	0.0056	0.0061	5.03	4.94E-07	3036.1	2910.94	3166.61
GGCGG	3496	4372	0.0051	0.0045	0.0043	0.0047	4.93	8.11E-07	3126.86	2999.72	3259.36
AGGGG	8348	10892	0.0193	0.0180	0.0176	0.0185	4.79	1.64E-06	7790.99	7590.1	7997.09
GGGGTG	3701	4658	0.0095	0.0085	0.0082	0.0089	4.78	1.78E-06	3333.2	3202.1	3469.62
CCCG	9158	11982	0.0176	0.0164	0.0160	0.0169	4.77	1.87E-06	8576.55	8365.49	8792.85
GTGGGG	4312	5473	0.0110	0.0100	0.0097	0.0104	4.75	2.01E-06	3916.4	3774.17	4063.93
CCGGG	3641	4585	0.0084	0.0076	0.0073	0.0079	4.73	2.25E-06	3279.63	3149.55	3415.04
GAGGG	9317	12214	0.0179	0.0168	0.0164	0.0172	4.71	2.50E-06	8737.74	8524.72	8955.99
CTGGG	13703	18210	0.0317	0.0302	0.0296	0.0308	4.55	5.24E-06	13025.5	12766.6	13289.5
GGGCC	9235	12132	0.0214	0.0201	0.0196	0.0206	4.55	5.29E-06	8677.95	8466.01	8895.09
GGGTGGG	2534	3142	0.0053	0.0047	0.0045	0.0050	4.52	6.20E-06	2246.8	2139.44	2359.52
GGGAGG	4992	6420	0.0097	0.0089	0.0086	0.0092	4.47	7.88E-06	4590.24	4435.96	4749.83
CAGG	26204	35335	0.0502	0.0485	0.0478	0.0492	4.45	8.39E-06	25292.3	24933.9	25655.7
GGCGGG	1832	2230	0.0035	0.0031	0.0029	0.0033	4.42	1.01E-05	1594.43	1504.33	1689.91
GGCCG	2938	3694	0.0057	0.0051	0.0049	0.0053	4.30	1.71E-05	2642.64	2526	2764.63
GGCAG	8855	11656	0.0171	0.0161	0.0157	0.0165	4.30	1.72E-05	8338.55	8130.44	8551.91
GCCC	24993	33742	0.0479	0.0463	0.0456	0.0470	4.20	2.66E-05	24152.1	23801.4	24507.6
GCAGG	8410	11078	0.0162	0.0153	0.0149	0.0157	4.14	3.48E-05	7925.06	7722.16	8133.21
CCCGG	3359	4274	0.0078	0.0071	0.0068	0.0074	4.10	4.13E-05	3057.17	2931.64	3188.04
GGGCA	7355	9656	0.0170	0.0160	0.0156	0.0164	4.10	4.20E-05	6906.88	6717.7	7101.31
GCCG	6440	8417	0.0093	0.0087	0.0084	0.0089	4.06	4.85E-05	6022.99	5845.87	6205.42
GGGGA	7934	10454	0.0184	0.0173	0.0169	0.0178	4.01	5.96E-05	7477.69	7280.87	7679.74
GGGGTGG	1932	2390	0.0050	0.0044	0.0042	0.0046	4.01	5.96E-05	1709.32	1616	1808.01
GGGGGC	2964	3760	0.0086	0.0078	0.0074	0.0081	3.97	7.24E-05	2689.89	2572.34	2812.77
CAGGG	10084	13391	0.0233	0.0222	0.0217	0.0227	3.95	7.95E-05	9578.51	9355.94	9806.25
GGCA	16420	22040	0.0315	0.0302	0.0297	0.0308	3.94	8.05E-05	15775.9	15490.7	16066.2
GGCAGG	3961	5098	0.0077	0.0071	0.0068	0.0073	3.94	8.13E-05	3645.02	3507.71	3787.67
GCAG	19909	26837	0.0287	0.0277	0.0272	0.0281	3.91	9.20E-05	19203.9	18888.5	19524.3
GGGCAG	4115	5305	0.0105	0.0097	0.0094	0.0101	3.90	9.51E-05	3796.18	3656.18	3941.5
GGGCGG	1694	2089	0.0033	0.0029	0.0027	0.0031	3.86	1.14E-04	1493.62	1406.49	1586.12
GGAGGG	4418	5729	0.0086	0.0079	0.0076	0.0082	3.79	1.48E-04	4096.18	3950.52	4247.17

TGGGGG	3851	4965	0.0111	0.0103	0.0099	0.0107	3.79	1.53E-04	3551.94	3416.63	3692.55
CGGGC	2639	3346	0.0051	0.0046	0.0044	0.0048	3.76	1.71E-04	2393.69	2282.78	2509.95
TGGGC	8072	10682	0.0187	0.0177	0.0172	0.0182	3.76	1.72E-04	7640.78	7441.83	7844.95
GGTGGGG	2137	2683	0.0055	0.0049	0.0047	0.0052	3.72	1.96E-04	1918.87	1819.87	2023.24
GGGCT	8563	11357	0.0198	0.0188	0.0183	0.0193	3.72	2.02E-04	8123.6	7918.49	8333.92
GCGGGG	1578	1946	0.0040	0.0036	0.0033	0.0038	3.70	2.15E-04	1392.53	1308.52	1481.91
TGGGGT	2774	3531	0.0071	0.0065	0.0062	0.0068	3.69	2.21E-04	2526.73	2412.82	2645.99
CGCG	2520	3193	0.0024	0.0022	0.0021	0.0023	3.69	2.22E-04	2284.24	2175.84	2398.03
CCCCG	3835	4954	0.0089	0.0082	0.0079	0.0085	3.69	2.23E-04	3543.57	3408.29	3684.17
CGGC	5893	7733	0.0085	0.0080	0.0077	0.0082	3.66	2.57E-04	5533.53	5363.82	5708.58
GGGGCT	3377	4345	0.0098	0.0090	0.0086	0.0094	3.63	2.83E-04	3108.4	2981.91	3240.2
GAGGGG	3255	4187	0.0083	0.0077	0.0073	0.0080	3.56	3.69E-04	2996.16	2871.95	3125.7
GGCCC	8552	11368	0.0198	0.0188	0.0183	0.0193	3.56	3.75E-04	8131.47	7926.26	8341.88
GGTGC	4477	5837	0.0104	0.0097	0.0093	0.0100	3.53	4.13E-04	4175.17	4028.22	4327.44
GGGGAG	3598	4655	0.0092	0.0085	0.0082	0.0089	3.49	4.85E-04	3331.05	3199.99	3467.43
CCGGGG	1298	1594	0.0037	0.0033	0.0031	0.0035	3.47	5.16E-04	1140.34	1064.55	1221.5
GCAGGG	3505	4533	0.0090	0.0083	0.0080	0.0086	3.46	5.41E-04	3243.75	3114.44	3378.38
CTGG	29860	40672	0.0572	0.0558	0.0551	0.0565	3.42	6.15E-04	29112.5	28729.2	29500.6
GCTGGG	4398	5746	0.0113	0.0105	0.0101	0.0109	3.38	7.30E-04	4111.76	3966	4262.81
AGGGC	7008	9294	0.0162	0.0154	0.0150	0.0158	3.36	7.77E-04	6647.95	6462.34	6838.8
GGGCAGG	1928	2434	0.0050	0.0045	0.0042	0.0047	3.36	7.81E-04	1740.79	1646.59	1840.35
GGGTGC	1922	2428	0.0056	0.0050	0.0047	0.0053	3.33	8.83E-04	1736.98	1642.91	1836.41
GCCCC	9070	12116	0.0210	0.0201	0.0196	0.0206	3.31	9.27E-04	8666.51	8454.71	8883.51
GCTGG	9013	12039	0.0174	0.0166	0.0162	0.0170	3.29	9.98E-04	8612.55	8401.05	8829.27
GCCGGG	1251	1543	0.0032	0.0028	0.0026	0.0030	3.29	1.00E-03	1104.15	1029.6	1184.08
CGGGT	1622	2034	0.0038	0.0034	0.0032	0.0036	3.27	1.06E-03	1454.91	1368.97	1546.22
GGGAGGG	2178	2775	0.0046	0.0042	0.0040	0.0044	3.26	1.11E-03	1984.36	1883.6	2090.49
CGGGGC	1382	1720	0.0035	0.0031	0.0029	0.0034	3.21	1.31E-03	1230.81	1151.97	1315.03
CCTGGG	5096	6715	0.0147	0.0139	0.0134	0.0143	3.20	1.37E-03	4803.88	4646.38	4966.65
CCCC	28534	38915	0.0140	0.0137	0.0135	0.0139	3.20	1.39E-03	27836.6	27453.8	28224.7
GGGTC	5090	6708	0.0118	0.0111	0.0107	0.0115	3.19	1.40E-03	4798.2	4640.57	4961.11
GGGCGGG	955	1162	0.0020	0.0017	0.0016	0.0019	3.19	1.41E-03	830.928	766.571	900.679
GGGCTG	4356	5713	0.0111	0.0105	0.0101	0.0108	3.17	1.51E-03	4088.14	3942.8	4238.78
GCCGG	2534	3258	0.0049	0.0045	0.0043	0.0047	3.17	1.55E-03	2330.73	2221.32	2445.5
CCCCGG	1248	1548	0.0036	0.0032	0.0030	0.0034	3.15	1.64E-03	1107.43	1032.78	1187.46
CCTGG	11975	16129	0.0277	0.0267	0.0261	0.0273	3.13	1.74E-03	11537	11293	11786.1
GCGC	4356	5719	0.0042	0.0039	0.0038	0.0041	3.12	1.78E-03	4091.32	3945.47	4242.53
GGAGC	5546	7337	0.0128	0.0121	0.0118	0.0125	3.12	1.79E-03	5248.12	5083.23	5418.28

TGGGGTG	1285	1599	0.0033	0.0029	0.0027	0.0031	3.12	1.82E-03	1143.6	1067.69	1224.89
CAGGGG	2747	3549	0.0079	0.0073	0.0070	0.0077	3.11	1.86E-03	2538.94	2424.79	2658.42
CCCGGG	1425	1785	0.0041	0.0037	0.0035	0.0039	3.09	1.97E-03	1276.98	1196.65	1362.69
CCGC	6284	8342	0.0091	0.0086	0.0083	0.0089	3.09	2.00E-03	5969.32	5793	6150.96
GGCCGG	1230	1530	0.0024	0.0021	0.0020	0.0023	3.07	2.17E-03	1093.94	1019.72	1173.54
GGGGGT	2208	2830	0.0064	0.0058	0.0056	0.0062	3.06	2.18E-03	2024.57	1922.84	2131.65
GGCTG	9896	13287	0.0191	0.0183	0.0179	0.0187	3.06	2.20E-03	9505.35	9283.22	9732.69
GGGCCG	1257	1565	0.0032	0.0029	0.0027	0.0031	3.06	2.25E-03	1119.89	1044.8	1200.37
GGGGGG	1769	2247	0.0009	0.0008	0.0008	0.0009	3.04	2.34E-03	1606.02	1515.49	1701.95
CTGGGG	4867	6424	0.0141	0.0133	0.0128	0.0137	3.04	2.37E-03	4595.7	4441.66	4755.01
GCGGGC	850	1034	0.0016	0.0014	0.0013	0.0016	3.02	2.54E-03	739.3	678.733	805.264
GTGGGGG	1347	1688	0.0039	0.0035	0.0033	0.0037	3.01	2.58E-03	1206.88	1128.85	1290.29
GGGGCC	3271	4265	0.0095	0.0088	0.0085	0.0092	3.01	2.63E-03	3051.16	2925.86	3181.78
GCCTGG	3678	4817	0.0094	0.0088	0.0085	0.0092	2.98	2.91E-03	3446.98	3313.63	3585.64
AGGC	16848	22850	0.0323	0.0313	0.0308	0.0319	2.97	3.00E-03	16355.7	16065.4	16651.1
GGCCGGG	655	783	0.0017	0.0014	0.0013	0.0016	2.96	3.03E-03	560	507.62	617.775
CGGGGG	1191	1484	0.0034	0.0031	0.0029	0.0033	2.96	3.06E-03	1061.65	988.607	1140.07
CGCGG	1074	1330	0.0025	0.0022	0.0020	0.0024	2.96	3.06E-03	951.342	882.311	1025.76
GGGGCTG	1810	2308	0.0053	0.0048	0.0045	0.0051	2.95	3.15E-03	1650.17	1558.53	1747.16
GGCAGGG	1699	2160	0.0044	0.0040	0.0037	0.0042	2.94	3.27E-03	1544.83	1456.22	1638.8
GGGGTC	1989	2547	0.0057	0.0053	0.0050	0.0056	2.94	3.31E-03	1822.11	1725.71	1923.87
GGGGCG	1384	1742	0.0035	0.0032	0.0030	0.0034	2.91	3.60E-03	1246.55	1167.19	1331.29
GGCGGGG	833	1018	0.0021	0.0019	0.0017	0.0020	2.89	3.89E-03	728.071	667.997	793.537
TGGGGC	3452	4522	0.0100	0.0093	0.0090	0.0097	2.89	3.89E-03	3235.02	3105.95	3369.4
GGGCCC	2982	3890	0.0086	0.0080	0.0077	0.0084	2.85	4.35E-03	2782.89	2663.3	2907.81
TGGGTG	2786	3628	0.0054	0.0050	0.0048	0.0053	2.84	4.47E-03	2593.99	2478.45	2714.88
GGGGAGG	1786	2284	0.0046	0.0042	0.0040	0.0044	2.83	4.62E-03	1633.51	1542.33	1730.06
GTGGGT	1837	2353	0.0036	0.0033	0.0031	0.0034	2.83	4.66E-03	1682.37	1589.76	1780.36
GGTC	10983	14813	0.0211	0.0203	0.0199	0.0208	2.83	4.71E-03	10602.9	10368.4	10842.7
GCCCCG	1269	1596	0.0032	0.0029	0.0027	0.0031	2.81	4.99E-03	1142.07	1066.22	1223.31
CGCC	5888	7845	0.0085	0.0081	0.0078	0.0083	2.78	5.46E-03	5613.68	5442.73	5789.95
GGGAGC	2305	2984	0.0067	0.0062	0.0059	0.0065	2.78	5.49E-03	2134.74	2030.23	2244.6
CGGGA	1869	2399	0.0043	0.0040	0.0038	0.0042	2.77	5.52E-03	1715.99	1622.46	1814.89
GGCTGG	4136	5465	0.0080	0.0076	0.0073	0.0078	2.77	5.61E-03	3907.42	3765.2	4054.98
TCGGG	1759	2254	0.0041	0.0037	0.0035	0.0040	2.74	6.07E-03	1612.27	1521.69	1708.23
GCCTGGG	1566	1998	0.0045	0.0041	0.0039	0.0044	2.73	6.35E-03	1428.52	1343.42	1518.99
CTGGGC	3698	4871	0.0095	0.0089	0.0086	0.0093	2.72	6.44E-03	3485.62	3351.52	3625.03
GTGGGTG	1010	1260	0.0021	0.0019	0.0018	0.0020	2.71	6.77E-03	901.007	833.888	973.517

TGGGAG	2968	3885	0.0086	0.0080	0.0077	0.0084	2.71	6.81E-03	2779.31	2659.8	2904.15
CCCCC	7047	9449	0.0036	0.0034	0.0033	0.0035	2.68	7.34E-03	6756.35	6568.12	6949.95
GGCCTGG	1614	2065	0.0041	0.0038	0.0036	0.0040	2.68	7.40E-03	1476.88	1390.3	1568.84
GCGCG	838	1035	0.0008	0.0007	0.0007	0.0008	2.67	7.56E-03	740.276	679.649	806.307
GAGGC	5825	7779	0.0135	0.0129	0.0125	0.0133	2.66	7.80E-03	5564.28	5394.48	5739.34
GGAGGGG	1641	2103	0.0042	0.0039	0.0036	0.0041	2.65	8.01E-03	1504.06	1416.66	1596.83
GAGGGC	2158	2797	0.0062	0.0058	0.0055	0.0061	2.65	8.16E-03	2000.96	1899.84	2107.43
TCGG	3732	4924	0.0072	0.0068	0.0065	0.0070	2.64	8.17E-03	3524.53	3389.53	3664.87
AGGGGG	1925	2484	0.0056	0.0051	0.0049	0.0054	2.64	8.25E-03	1777.04	1681.87	1877.57
GGGCCGG	595	719	0.0015	0.0013	0.0012	0.0015	2.64	8.37E-03	514.227	464.138	569.713
GGGGCA	2491	3247	0.0072	0.0067	0.0064	0.0070	2.63	8.46E-03	2322.89	2213.78	2437.34
GGGGTGC	692	848	0.0023	0.0020	0.0018	0.0022	2.58	9.81E-03	606.39	551.793	666.376
GGTCC	4124	5469	0.0095	0.0091	0.0087	0.0094	2.57	1.01E-02	3911.95	3769.74	4059.47
CCCGC	2782	3647	0.0054	0.0050	0.0048	0.0053	2.56	1.05E-02	2609.02	2493.14	2730.25
CGGCC	2392	3120	0.0046	0.0043	0.0041	0.0045	2.55	1.07E-02	2232.01	2124.99	2344.39
GGCT	20057	27378	0.0384	0.0376	0.0370	0.0382	2.55	1.09E-02	19596.8	19279.8	19918.8
GCGGGGC	576	698	0.0015	0.0013	0.0012	0.0014	2.55	1.09E-02	499.208	449.893	553.921
GGAGGC	2497	3263	0.0072	0.0067	0.0064	0.0071	2.54	1.10E-02	2334.34	2224.96	2449.05
GTGC	11323	15334	0.0217	0.0210	0.0206	0.0215	2.54	1.11E-02	10975.9	10737.3	11219.6
GGGGGTG	1180	1496	0.0034	0.0031	0.0029	0.0033	2.53	1.15E-02	1069.6	996.283	1148.31
TGGGCG	803	996	0.0023	0.0021	0.0019	0.0022	2.52	1.16E-02	712.534	653.137	777.321
GGCTGGG	2117	2754	0.0054	0.0051	0.0048	0.0053	2.50	1.23E-02	1969.65	1869.31	2075.35
GCAGGGG	1110	1405	0.0032	0.0029	0.0027	0.0031	2.49	1.27E-02	1004.54	933.559	1080.91
TGGGGGC	1206	1533	0.0040	0.0036	0.0034	0.0039	2.49	1.29E-02	1096.22	1021.98	1175.84
GCGGGT	558	677	0.0016	0.0014	0.0013	0.0016	2.48	1.31E-02	484.323	435.79	538.253
GAGGCA	1432	1835	0.0041	0.0038	0.0036	0.0040	2.47	1.35E-02	1312.75	1231.27	1399.61
TGGGA	6512	8746	0.0151	0.0145	0.0141	0.0149	2.47	1.35E-02	6255.97	6075.91	6441.28
TGGGT	5675	7599	0.0109	0.0105	0.0101	0.0108	2.46	1.38E-02	5436.23	5268.23	5609.52
GGGAGGC	1034	1306	0.0034	0.0031	0.0029	0.0033	2.45	1.42E-02	933.898	865.554	1007.62
CGGGGT	747	926	0.0022	0.0019	0.0017	0.0021	2.45	1.44E-02	662.457	605.273	725.031
AGGGGC	2690	3533	0.0078	0.0073	0.0070	0.0076	2.44	1.45E-02	2527.49	2413.6	2646.72
GCCCCC	2438	3193	0.0070	0.0066	0.0063	0.0069	2.43	1.51E-02	2284.26	2176.08	2397.78
GGTGGGT	818	1021	0.0017	0.0015	0.0014	0.0017	2.43	1.53E-02	730.102	669.931	795.667
GTGGGGT	809	1009	0.0021	0.0019	0.0017	0.0020	2.43	1.53E-02	721.634	661.837	786.823
CGAG	3704	4910	0.0071	0.0067	0.0065	0.0070	2.42	1.55E-02	3514.51	3379.71	3654.65
CCAGG	9570	12955	0.0222	0.0215	0.0209	0.0220	2.42	1.57E-02	9266.64	9047.69	9490.77
CGTGG	2172	2835	0.0050	0.0047	0.0045	0.0049	2.41	1.58E-02	2027.86	1925.99	2135.09
TGGGTGG	1253	1600	0.0026	0.0024	0.0022	0.0026	2.41	1.58E-02	1144.14	1068.19	1225.47

GGGTGCA	484	584	0.0016	0.0014	0.0012	0.0016	2.40	1.62E-02	417.608	372.723	467.89
GGCCAG	2654	3487	0.0068	0.0064	0.0061	0.0067	2.40	1.63E-02	2495.25	2382.05	2613.78
GCGGC	1930	2510	0.0028	0.0026	0.0025	0.0027	2.40	1.66E-02	1795.15	1699.37	1896.32
AGGTG	4976	6653	0.0115	0.0110	0.0107	0.0114	2.39	1.66E-02	4758.86	4601.89	4921.12
ACGG	3001	3958	0.0058	0.0054	0.0052	0.0057	2.39	1.70E-02	2833.08	2712.24	2959.27
GTGGGC	2358	3089	0.0068	0.0064	0.0061	0.0067	2.38	1.73E-02	2209.86	2103.49	2321.57
CGTGGG	951	1199	0.0027	0.0025	0.0023	0.0027	2.38	1.73E-02	857.76	792.351	928.553
CCGCC	2671	3514	0.0039	0.0036	0.0035	0.0038	2.38	1.75E-02	2513.22	2399.46	2632.34
CCCCGGG	527	641	0.0017	0.0015	0.0014	0.0017	2.38	1.75E-02	458.368	411.225	510.906
GGCGC	1718	2226	0.0040	0.0037	0.0035	0.0039	2.37	1.77E-02	1592.25	1502.24	1687.63
CGAGG	1726	2237	0.0040	0.0037	0.0035	0.0039	2.37	1.78E-02	1600.11	1509.88	1695.72
GCCGGGG	479	579	0.0014	0.0012	0.0011	0.0013	2.37	1.80E-02	413.971	369.29	464.052
GGGGCGG	743	925	0.0019	0.0017	0.0016	0.0019	2.36	1.83E-02	661.558	604.409	724.099
CGGGTG	793	991	0.0023	0.0020	0.0019	0.0022	2.36	1.85E-02	708.957	649.716	773.589
GGGTCC	1902	2475	0.0055	0.0051	0.0048	0.0054	2.35	1.86E-02	1770.6	1675.61	1870.96
GGTGGGC	1071	1361	0.0036	0.0032	0.0030	0.0035	2.35	1.88E-02	973.227	903.409	1048.42
TGCGG	1588	2053	0.0037	0.0034	0.0032	0.0036	2.35	1.90E-02	1468.5	1382.15	1560.22
GCCTG	8288	11202	0.0160	0.0154	0.0150	0.0158	2.34	1.92E-02	8013.77	7809.73	8223.05
CGGCG	906	1142	0.0013	0.0012	0.0011	0.0013	2.33	1.96E-02	816.759	752.959	885.959
GGAGCC	2060	2690	0.0060	0.0056	0.0053	0.0059	2.33	1.97E-02	1924.41	1825.29	2028.89
TGCGGG	692	859	0.0020	0.0018	0.0016	0.0019	2.33	1.99E-02	614.525	559.541	674.902
CTGGGAG	1169	1495	0.0039	0.0035	0.0033	0.0038	2.29	2.18E-02	1069.05	995.76	1147.71
GGCGGGC	420	505	0.0009	0.0008	0.0007	0.0009	2.29	2.20E-02	361.118	319.546	408.093
GTGTG	4979	6671	0.0050	0.0048	0.0046	0.0050	2.28	2.26E-02	4771.38	4613.73	4934.39
CCCGCC	1298	1669	0.0025	0.0023	0.0022	0.0025	2.28	2.29E-02	1193.32	1115.7	1276.33
CGCGGG	442	534	0.0013	0.0011	0.0010	0.0012	2.27	2.31E-02	382.021	339.197	430.245
GGGGGCT	916	1159	0.0030	0.0027	0.0025	0.0030	2.27	2.34E-02	828.78	764.538	898.405
GCTGGGC	1140	1458	0.0029	0.0027	0.0025	0.0029	2.26	2.39E-02	1042.76	970.381	1120.52
GGCCCC	3015	3992	0.0087	0.0083	0.0079	0.0086	2.26	2.40E-02	2855.86	2734.69	2982.36
GGGCGC	741	927	0.0021	0.0019	0.0018	0.0021	2.26	2.41E-02	663.172	605.956	725.779
GGGCTGG	1949	2547	0.0050	0.0047	0.0044	0.0049	2.25	2.43E-02	1821.61	1725.2	1923.38
GGGGCCG	506	619	0.0015	0.0013	0.0012	0.0014	2.24	2.52E-02	442.571	396.287	494.252
ATGGG	4165	5564	0.0096	0.0092	0.0089	0.0096	2.23	2.58E-02	3979.9	3836.45	4128.66
CGGGCG	436	528	0.0008	0.0007	0.0006	0.0008	2.23	2.58E-02	377.515	334.953	425.481
GAGGGGG	781	982	0.0023	0.0020	0.0019	0.0022	2.22	2.61E-02	702.107	643.164	766.439
CGCCC	2418	3184	0.0047	0.0044	0.0042	0.0046	2.22	2.64E-02	2277.79	2169.66	2391.29
GGAGGCA	663	826	0.0022	0.0020	0.0018	0.0022	2.22	2.65E-02	590.658	536.807	649.9
GGACG	1146	1468	0.0022	0.0020	0.0019	0.0022	2.22	2.66E-02	1050.19	977.523	1128.24

GCCCG	2229	2928	0.0043	0.0040	0.0038	0.0042	2.22	2.67E-02	2094.65	1991.05	2203.63
CTGGGGT	1105	1414	0.0037	0.0034	0.0031	0.0036	2.22	2.67E-02	1011.13	939.916	1087.71
CCTG	30490	41914	0.0584	0.0575	0.0568	0.0583	2.21	2.70E-02	30001.5	29612.7	30395.1
GGGGCAG	1396	1805	0.0041	0.0037	0.0035	0.0040	2.21	2.72E-02	1290.53	1209.76	1376.68
TGGGCC	2592	3422	0.0075	0.0071	0.0067	0.0074	2.20	2.77E-02	2448.08	2336.03	2565.48
AGGCAG	2341	3083	0.0045	0.0043	0.0041	0.0045	2.20	2.78E-02	2204.32	2097.98	2316.02
GCTGGGG	1680	2190	0.0049	0.0045	0.0043	0.0048	2.18	2.95E-02	1565.8	1476.6	1660.36
GTGGGGC	1098	1407	0.0036	0.0033	0.0031	0.0036	2.17	2.97E-02	1006.12	935.093	1082.52
CCGGC	2229	2932	0.0043	0.0040	0.0038	0.0042	2.17	3.01E-02	2097.52	1993.84	2206.56
GAGGGGC	1079	1382	0.0036	0.0033	0.0030	0.0035	2.17	3.02E-02	988.244	917.871	1063.99
TGGGCGG	372	447	0.0012	0.0011	0.0009	0.0012	2.16	3.04E-02	319.642	280.685	363.999
AGGGAG	2733	3619	0.0053	0.0050	0.0048	0.0052	2.16	3.05E-02	2587.55	2472.16	2708.3
AGGCC	6151	8294	0.0142	0.0137	0.0133	0.0142	2.16	3.05E-02	5932.65	5757.32	6113.25
GGCTGTG	1006	1285	0.0029	0.0027	0.0025	0.0029	2.16	3.09E-02	918.745	850.966	991.907
AGGCA	4153	5556	0.0080	0.0077	0.0074	0.0079	2.15	3.17E-02	3974.69	3831.23	4123.48
GTCGG	997	1273	0.0019	0.0018	0.0016	0.0019	2.14	3.20E-02	910.688	843.191	983.578
ATGGGG	1705	2225	0.0049	0.0046	0.0043	0.0049	2.14	3.23E-02	1591.76	1501.8	1687.08
AGGGCG	690	865	0.0020	0.0018	0.0016	0.0020	2.14	3.27E-02	618.817	563.633	679.395
CCGCG	1010	1291	0.0023	0.0021	0.0020	0.0023	2.14	3.27E-02	923.445	855.471	996.809
GAGC	13234	18049	0.0254	0.0248	0.0243	0.0253	2.13	3.31E-02	12919.2	12660.7	13182.9
GGCCAGG	1293	1671	0.0033	0.0031	0.0029	0.0033	2.13	3.32E-02	1195.1	1117.44	1278.13
GCCAGG	2812	3727	0.0072	0.0068	0.0065	0.0071	2.13	3.34E-02	2666.99	2549.9	2789.41
CCCCCG	1038	1329	0.0030	0.0027	0.0025	0.0030	2.12	3.38E-02	950.761	881.769	1025.14
AGGCAGG	1048	1343	0.0022	0.0020	0.0019	0.0022	2.12	3.39E-02	960.359	890.984	1035.12
GGCGCG	348	417	0.0009	0.0008	0.0007	0.0009	2.12	3.39E-02	298.399	260.84	341.362
GGGGGA	1885	2470	0.0054	0.0051	0.0048	0.0054	2.12	3.41E-02	1767.03	1672.13	1867.28
GGCCGC	775	979	0.0022	0.0020	0.0019	0.0022	2.11	3.49E-02	700.373	641.506	764.63
CAGGGC	3154	4195	0.0081	0.0077	0.0074	0.0080	2.11	3.52E-02	3001.88	2877.55	3131.54
GGCCCG	799	1011	0.0020	0.0019	0.0017	0.0020	2.10	3.56E-02	723.457	663.581	788.725
GCCCCGG	431	526	0.0013	0.0011	0.0010	0.0012	2.10	3.57E-02	376.078	333.608	423.948
GCGGGCG	214	246	0.0005	0.0004	0.0003	0.0004	2.10	3.57E-02	175.911	147.648	209.582
GCCCTG	3612	4821	0.0092	0.0088	0.0085	0.0092	2.10	3.60E-02	3449.84	3316.44	3588.56
CCGGGA	675	847	0.0020	0.0018	0.0016	0.0019	2.09	3.62E-02	605.94	551.359	665.915
TGCG	3774	5041	0.0072	0.0069	0.0067	0.0072	2.09	3.63E-02	3608.28	3471.67	3750.23
TGGGAGG	1311	1698	0.0043	0.0040	0.0038	0.0043	2.09	3.65E-02	1214.21	1135.95	1297.84
CGGGAG	874	1112	0.0025	0.0023	0.0021	0.0025	2.08	3.71E-02	795.52	732.62	863.808
AGGGA	5946	8022	0.0115	0.0111	0.0107	0.0114	2.08	3.71E-02	5738.84	5566.21	5916.76
CCTGGGG	1738	2275	0.0058	0.0054	0.0051	0.0057	2.08	3.74E-02	1626.81	1535.87	1723.1

AGGGGA	1960	2574	0.0050	0.0047	0.0045	0.0050	2.08	3.77E-02	1841.92	1744.96	1944.24
AGGT	10087	13717	0.0193	0.0188	0.0184	0.0193	2.08	3.78E-02	9818.44	9592.7	10049.4
CGGAG	1508	1964	0.0035	0.0033	0.0031	0.0035	2.07	3.81E-02	1404.84	1320.43	1494.62
GGGATG	1644	2147	0.0042	0.0039	0.0037	0.0042	2.07	3.84E-02	1536.36	1448	1630.09
GGGGGCG	476	586	0.0014	0.0012	0.0011	0.0014	2.07	3.84E-02	418.976	374.011	469.341
GTGGGCG	338	406	0.0010	0.0008	0.0007	0.0010	2.07	3.85E-02	290.28	253.271	332.692
GACGG	1023	1312	0.0020	0.0018	0.0017	0.0020	2.07	3.87E-02	938.588	870.027	1012.54
GCGCGC	268	316	0.0003	0.0002	0.0002	0.0003	2.06	3.93E-02	225.884	193.525	263.653
GCCGC	1826	2395	0.0026	0.0025	0.0023	0.0026	2.06	3.93E-02	1712.91	1619.4	1811.8
CCCGCCC	626	784	0.0013	0.0012	0.0011	0.0013	2.06	3.94E-02	560.626	508.21	618.442
GGTGCA	989	1267	0.0029	0.0026	0.0024	0.0028	2.06	3.95E-02	906.407	839.1	979.096
CGGCGG	384	466	0.0006	0.0005	0.0005	0.0006	2.06	3.96E-02	333.269	293.428	378.516
GGACGG	468	576	0.0009	0.0008	0.0007	0.0009	2.06	3.97E-02	411.835	367.268	461.805
TGGGCA	2163	2852	0.0062	0.0059	0.0056	0.0062	2.05	3.99E-02	2040.31	1938.18	2147.79
AGGGAGG	1345	1746	0.0028	0.0026	0.0025	0.0028	2.05	3.99E-02	1248.54	1169.09	1333.37
CCCCCGG	323	387	0.0011	0.0009	0.0008	0.0011	2.05	4.00E-02	276.737	240.662	318.214
GGCCTG	3246	4326	0.0083	0.0079	0.0076	0.0083	2.05	4.02E-02	3095.62	2969.34	3227.23
GTGTGTG	843	1074	0.0009	0.0008	0.0007	0.0009	2.04	4.14E-02	767.535	705.756	834.717
AGGGGT	1646	2153	0.0048	0.0044	0.0042	0.0047	2.03	4.20E-02	1540.25	1451.79	1634.06
GGTGCC	1643	2149	0.0047	0.0044	0.0042	0.0047	2.03	4.21E-02	1537.39	1449.02	1631.12
GAGGCAG	846	1078	0.0025	0.0022	0.0021	0.0024	2.03	4.22E-02	770.745	708.87	838.007
TGGCC	6637	8981	0.0154	0.0149	0.0144	0.0153	2.03	4.24E-02	6424.06	6241.6	6611.77
TGGGCCC	923	1181	0.0031	0.0028	0.0026	0.0030	2.03	4.27E-02	844.512	779.64	914.766
TGGGGGT	984	1263	0.0029	0.0026	0.0024	0.0028	2.02	4.30E-02	903.015	835.84	975.574
CCGAG	1595	2085	0.0037	0.0035	0.0033	0.0037	2.02	4.31E-02	1491.39	1404.35	1583.8
CGGCCG	373	453	0.0007	0.0006	0.0006	0.0007	2.02	4.32E-02	323.891	284.652	368.534
TGTGGG	2499	3312	0.0072	0.0068	0.0065	0.0072	2.02	4.37E-02	2369.39	2259.18	2484.94
CCGCCC	1173	1517	0.0023	0.0021	0.0020	0.0023	2.02	4.37E-02	1084.64	1010.75	1163.92
GGCCCCG	476	588	0.0014	0.0012	0.0011	0.0014	2.02	4.37E-02	420.406	375.36	470.851
CCCCGCC	657	827	0.0017	0.0015	0.0014	0.0017	2.01	4.41E-02	591.468	537.568	650.764
GGAGGGC	968	1242	0.0032	0.0029	0.0027	0.0032	2.01	4.42E-02	888.132	821.544	960.101
GAGCC	5001	6736	0.0116	0.0112	0.0108	0.0115	2.01	4.48E-02	4818.22	4660.27	4981.47
GGAGGA	2176	2875	0.0034	0.0032	0.0030	0.0033	2.00	4.57E-02	2056.11	1953.45	2164.15
GCTG	22853	31397	0.0329	0.0324	0.0319	0.0329	1.99	4.63E-02	22466.9	22126.4	22812.4
ACGGG	1257	1631	0.0029	0.0027	0.0025	0.0029	1.99	4.65E-02	1166.65	1089.94	1248.73
TTGGG	4745	6387	0.0110	0.0106	0.0102	0.0109	1.99	4.68E-02	4568.59	4414.81	4727.66
TGGGCAG	1145	1481	0.0038	0.0035	0.0033	0.0038	1.99	4.69E-02	1059.04	986.104	1137.34
CGGGGA	847	1081	0.0024	0.0022	0.0021	0.0024	1.99	4.71E-02	773.343	711.36	840.714

CGTG	5155	6945	0.0099	0.0095	0.0092	0.0098	1.99	4.71E-02	4971.14	4810.53	5137.05
CCCGGC	1016	1307	0.0026	0.0024	0.0022	0.0026	1.98	4.74E-02	935.271	866.853	1009.07
GTCG	2163	2857	0.0031	0.0029	0.0028	0.0031	1.98	4.75E-02	2044.39	1942.02	2152.15
CGGGCC	850	1085	0.0022	0.0020	0.0018	0.0022	1.98	4.77E-02	776.411	714.293	843.919
GGCCA	5599	7561	0.0130	0.0125	0.0121	0.0129	1.98	4.80E-02	5408.34	5240.95	5581.01
AGGCCG	619	778	0.0018	0.0016	0.0015	0.0018	1.98	4.81E-02	556.578	504.371	614.18
GCGAG	1078	1391	0.0021	0.0019	0.0018	0.0021	1.97	4.83E-02	995.104	924.435	1071.16
CGGGTC	434	534	0.0011	0.0010	0.0009	0.0011	1.97	4.86E-02	382.123	339.29	430.356
CGCCG	853	1090	0.0012	0.0011	0.0010	0.0012	1.97	4.87E-02	779.569	717.296	847.241
CCCGGGG	480	595	0.0016	0.0014	0.0013	0.0016	1.97	4.91E-02	425.474	380.145	476.199
AGGGCAG	1125	1455	0.0029	0.0027	0.0025	0.0029	1.97	4.92E-02	1040.61	968.312	1118.3
AGAGGG	2022	2668	0.0058	0.0055	0.0052	0.0058	1.96	4.98E-02	1908.68	1809.96	2012.74
TGGGGA	3033	4046	0.0088	0.0084	0.0080	0.0087	1.95	5.06E-02	2894.49	2772.49	3021.81
GCCAG	6093	8242	0.0117	0.0114	0.0110	0.0117	1.95	5.06E-02	5896.22	5721.23	6076.5
CCCCCC	1427	1864	0.0007	0.0007	0.0006	0.0007	1.95	5.07E-02	1332.27	1250.05	1419.9
TGTGG	5950	8047	0.0138	0.0133	0.0129	0.0137	1.95	5.09E-02	5755.98	5583.28	5933.94
AGGGGCA	675	854	0.0020	0.0018	0.0016	0.0019	1.95	5.12E-02	610.59	555.79	670.782
AGGGGAG	926	1189	0.0024	0.0022	0.0020	0.0024	1.95	5.16E-02	850.37	785.246	920.882
AGGTGGG	1164	1509	0.0039	0.0036	0.0033	0.0038	1.95	5.16E-02	1079.06	1005.42	1158.07
TGGAGG	2409	3196	0.0070	0.0066	0.0063	0.0069	1.94	5.21E-02	2286.41	2178.17	2399.98
GGGAC	4488	6040	0.0104	0.0100	0.0097	0.0104	1.94	5.22E-02	4320.38	4170.87	4475.2
GGTCGG	429	529	0.0008	0.0007	0.0006	0.0008	1.94	5.23E-02	378.23	335.625	426.239
TGGGGCT	1149	1490	0.0033	0.0031	0.0029	0.0033	1.93	5.37E-02	1065.32	992.145	1143.86
CAGGC	6792	9208	0.0131	0.0127	0.0123	0.0131	1.93	5.41E-02	6587.28	6402.29	6777.55
GTGCCCC	549	688	0.0018	0.0016	0.0015	0.0018	1.92	5.50E-02	491.977	443.046	546.301
CGGGGGC	407	501	0.0012	0.0010	0.0009	0.0012	1.92	5.53E-02	358.203	316.815	404.992
GGCTGCG	312	377	0.0009	0.0008	0.0007	0.0009	1.91	5.57E-02	269.546	233.974	310.522
TGGGGTC	763	973	0.0025	0.0023	0.0021	0.0025	1.91	5.61E-02	695.775	637.117	759.82
TGAGG	5268	7115	0.0122	0.0118	0.0114	0.0122	1.91	5.61E-02	5089.32	4926.96	5256.96
GTCGGG	509	635	0.0013	0.0012	0.0010	0.0013	1.91	5.62E-02	454.397	407.462	506.731
CCGCGG	423	522	0.0012	0.0011	0.0010	0.0012	1.91	5.65E-02	373.437	331.125	421.149
CGGGGTG	351	428	0.0012	0.0010	0.0009	0.0012	1.91	5.67E-02	306.055	267.989	349.522
GCGGGTG	301	363	0.0009	0.0008	0.0007	0.0009	1.90	5.69E-02	259.537	224.678	299.799
GCGGGGG	450	558	0.0013	0.0012	0.0010	0.0013	1.90	5.71E-02	398.957	355.139	448.174
GGCGT	1264	1646	0.0029	0.0027	0.0025	0.0029	1.90	5.73E-02	1177.37	1100.31	1259.83
GGCAGGC	836	1071	0.0018	0.0016	0.0015	0.0018	1.90	5.73E-02	765.856	704.169	832.937
TGTGGGG	965	1244	0.0032	0.0029	0.0027	0.0032	1.90	5.73E-02	889.562	822.919	961.586
CGGGGCC	503	628	0.0015	0.0013	0.0012	0.0015	1.90	5.74E-02	449.005	402.368	501.04

GGTGAG	1564	2051	0.0040	0.0038	0.0035	0.0040	1.90	5.77E-02	1467.67	1381.36	1559.35
CCGGGGA	273	327	0.0009	0.0008	0.0007	0.0009	1.89	5.85E-02	233.832	200.872	272.196
GGGCGCG	177	204	0.0005	0.0004	0.0003	0.0005	1.89	5.91E-02	145.855	120.34	176.778
TGGGGGG	804	1029	0.0027	0.0024	0.0022	0.0027	1.89	5.93E-02	735.82	675.428	801.597
GCCCGG	936	1205	0.0024	0.0022	0.0020	0.0024	1.89	5.94E-02	862.281	796.685	933.264
GGGCCTG	1281	1671	0.0037	0.0035	0.0032	0.0037	1.88	5.99E-02	1194.73	1117.1	1277.73
CAGGTG	1968	2601	0.0057	0.0054	0.0051	0.0057	1.88	5.99E-02	1860.74	1763.31	1963.54
GTGGC	5159	6969	0.0119	0.0115	0.0112	0.0119	1.88	6.01E-02	4984.89	4824.21	5150.85
GCGAGG	527	660	0.0013	0.0012	0.0011	0.0013	1.88	6.03E-02	472.287	424.388	525.584
TGGGGAG	1346	1759	0.0045	0.0042	0.0039	0.0045	1.88	6.08E-02	1257.83	1178.14	1342.89
CCGGGGG	331	403	0.0011	0.0010	0.0008	0.0011	1.87	6.15E-02	288.178	251.314	330.444
GGGATGG	784	1003	0.0020	0.0018	0.0017	0.0020	1.87	6.20E-02	717.343	657.731	782.347
TGGC	17103	23457	0.0328	0.0322	0.0316	0.0328	1.87	6.20E-02	16790.2	16496.2	17089.3
GCGCGG	357	437	0.0009	0.0008	0.0007	0.0009	1.86	6.31E-02	312.711	274.202	356.623
CCCTG	11214	15325	0.0260	0.0254	0.0248	0.0259	1.85	6.38E-02	10961.9	10724	11204.9
GGGAGCC	848	1090	0.0028	0.0026	0.0024	0.0028	1.84	6.52E-02	779.44	717.212	847.051
GGATGGG	822	1055	0.0021	0.0019	0.0018	0.0021	1.84	6.53E-02	754.533	693.332	821.125
GGGGGCC	783	1003	0.0026	0.0024	0.0022	0.0026	1.84	6.54E-02	717.228	657.635	782.206
CCGGGC	1022	1323	0.0026	0.0024	0.0022	0.0026	1.84	6.58E-02	946.72	877.87	1020.96
AGGGGTG	773	990	0.0026	0.0023	0.0022	0.0026	1.83	6.65E-02	707.932	648.742	772.507
CGCGC	750	959	0.0008	0.0007	0.0006	0.0008	1.83	6.67E-02	685.918	627.654	749.586
GTGGGGA	996	1290	0.0033	0.0031	0.0028	0.0033	1.82	6.85E-02	922.456	854.547	995.744
CTGGGA	2631	3510	0.0076	0.0073	0.0069	0.0076	1.82	6.93E-02	2511.04	2397.53	2629.89
GTGCCC	1683	2219	0.0049	0.0046	0.0043	0.0049	1.81	6.99E-02	1587.46	1497.63	1682.66
