# Supplementary material for: Identification of motifs that function in the splicing of non-canonical introns
Source: Genome Biol. 2008 Jun 12;9(6):R97. doi: 10.1186/gb-2008-9-6-r97 (PMC2481429; doi:10.1186/gb-2008-9-6-r97)
Supplement: Additional data file 3 — Associated statistics and listing of n-mers (4-7 nucleotides) determined to be enriched in the 50 nucleotide region upstream of weak PY tracts from AT-rich introns. [file gb-2008-9-6-r97-S3.pdf]

Nmers enriched upstream of weak PY tracts for AT-rich introns

**Field**	**Description**
Nmer	The Nmer evaluated
Count(S)	The observed counts for the nmer in the sample
Count(P)	The observed counts for the nmer in the background
Prob(S)	Probability of occurrence for the nmer in the sample
Prob(P)	Probability of occurrence for the nmer in the background
CI(low)	Lower confidence interval (P<0.01) based upon binomial distribution
CI(high)	Upper confidence interval (P<0.01) based upon binomial distribution
Z_score	Z score (based upon binomial distribution)
P(Z)	Probability of observation
E(S)	Expected occurrence in sample (based upon sample size and background probabilities)
E(CI_low)	Minimal occurrence in sample expected by chance (based upon sample size and lower CI)
E(CI_high)	Maximum occurrence in sample expected by chance (based upon sample size and upper CI)

Nmer	Count(S)	Count(P)	Prob(S)	Prob(P)	CI(low)	CI(high)	Z_score	P(Z)	E(S)	E(CI_low)	E(CI_high)
GGGG	4240	10397	0.0021	0.0019	0.0018	0.0020	5.44	5.28E-08	3840.28	3698.92	3987.02
TGGG	7385	18666	0.0144	0.0134	0.0130	0.0138	5.03	4.97E-07	6895.18	6705.92	7089.7
GGGA	6088	15444	0.0118	0.0111	0.0108	0.0114	4.32	1.57E-05	5704.98	5532.87	5882.38
TGTG	14356	37278	0.0140	0.0134	0.0132	0.0137	4.28	1.87E-05	13769	13500.5	14042.8
TTGGG	2360	5877	0.0055	0.0051	0.0048	0.0053	3.44	5.78E-04	2170.66	2065.2	2281.48
GGGT	4579	11704	0.0089	0.0084	0.0081	0.0087	3.31	9.35E-04	4323.43	4173.75	4478.44
TGGGG	1858	4598	0.0043	0.0040	0.0038	0.0042	3.28	1.05E-03	1698.27	1605.23	1796.67
GGGGG	924	2203	0.0005	0.0004	0.0004	0.0004	3.25	1.16E-03	813.648	749.952	882.75
AGGG	5312	13661	0.0103	0.0098	0.0095	0.0101	3.19	1.43E-03	5046.34	4884.53	5213.46
GTGTG	3251	8245	0.0033	0.0031	0.0030	0.0032	3.16	1.58E-03	3045.43	2919.91	3176.33
GTGT	10775	28156	0.0105	0.0101	0.0099	0.0104	3.15	1.66E-03	10399.7	10166.3	10638.4
GTGC	4546	11666	0.0088	0.0084	0.0081	0.0087	3.07	2.14E-03	4309.4	4159.96	4464.15
GGGGA	1508	3720	0.0035	0.0032	0.0030	0.0034	3.05	2.25E-03	1373.98	1290.53	1462.8
TGAG	9029	23557	0.0176	0.0169	0.0165	0.0174	3.01	2.64E-03	8701.91	8489.34	8919.7
TGGGGG	452	1035	0.0013	0.0011	0.0010	0.0013	2.98	2.92E-03	382.298	339.458	430.538
TGTGC	2137	5363	0.0050	0.0046	0.0044	0.0049	2.97	2.93E-03	1980.82	1880.17	2086.83
TGTGCA	704	1669	0.0021	0.0018	0.0016	0.0020	2.96	3.09E-03	616.478	561.403	676.946
TGTGT	5752	14883	0.0058	0.0056	0.0054	0.0058	2.93	3.44E-03	5497.29	5327.95	5671.99
TGTGTG	1949	4882	0.0020	0.0018	0.0017	0.0019	2.91	3.61E-03	1802.95	1706.92	1904.37
CTGTG	3315	8459	0.0078	0.0073	0.0070	0.0076	2.90	3.70E-03	3124.32	2997.4	3256.57
GTGTGT	1697	4231	0.0017	0.0016	0.0015	0.0017	2.88	4.02E-03	1562.53	1473.3	1657.15

AGGGG	1188	2917	0.0028	0.0025	0.0023	0.0027	2.84	4.45E-03	1077.39	1003.77	1156.39
TGCA	8427	22020	0.0164	0.0158	0.0154	0.0162	2.78	5.37E-03	8134.14	7928.6	8344.92
GGGGT	1092	2676	0.0026	0.0023	0.0021	0.0025	2.78	5.42E-03	988.377	917.969	1064.17
TGGGGGT	136	276	0.0004	0.0003	0.0002	0.0004	2.76	5.75E-03	101.935	81.0004	128.278
GAGG	5356	13875	0.0078	0.0075	0.0072	0.0077	2.75	6.01E-03	5125.32	4962.04	5293.93
ACACAC	615	1460	0.0006	0.0005	0.0005	0.0006	2.74	6.15E-03	539.185	487.817	595.96
TGAGG	1897	4774	0.0044	0.0041	0.0039	0.0044	2.70	6.94E-03	1763.27	1668.43	1863.48
GGACAGA	130	264	0.0004	0.0003	0.0003	0.0004	2.70	7.04E-03	97.4963	77.0753	123.326
AAGGGG	393	905	0.0011	0.0010	0.0009	0.0011	2.68	7.29E-03	334.28	294.382	379.578
GAGT	6647	17317	0.0129	0.0124	0.0121	0.0128	2.68	7.46E-03	6396.86	6214.58	6584.42
TGCAA	2149	5437	0.0050	0.0047	0.0045	0.0049	2.67	7.65E-03	2008.15	1906.79	2114.87
CTTG	9573	25109	0.0186	0.0180	0.0176	0.0185	2.66	7.93E-03	9275.21	9055.79	9499.85
GGGAT	1367	3400	0.0032	0.0029	0.0028	0.0031	2.65	7.94E-03	1255.79	1176.11	1340.84
GCTG	6589	17173	0.0096	0.0093	0.0090	0.0095	2.63	8.49E-03	6343.57	6161.78	6530.67
GAGGG	1316	3270	0.0026	0.0024	0.0022	0.0025	2.63	8.51E-03	1207.84	1129.74	1291.34
GAGTTGC	115	231	0.0004	0.0003	0.0002	0.0004	2.63	8.61E-03	85.3093	66.3605	109.667
CTTGAGG	135	278	0.0005	0.0003	0.0003	0.0004	2.62	8.83E-03	102.667	81.6487	129.093
GGTG	5336	13854	0.0078	0.0075	0.0072	0.0077	2.60	9.21E-03	5117.56	4954.41	5286.04
CCTGTG	689	1658	0.0020	0.0018	0.0016	0.0020	2.60	9.23E-03	612.415	557.53	672.693
GGGGGT	256	571	0.0007	0.0006	0.0005	0.0007	2.58	9.86E-03	210.91	179.731	247.495
GAAC	5038	13085	0.0098	0.0094	0.0091	0.0097	2.51	1.21E-02	4833.57	4675.23	4997.22
TTGG	10030	26372	0.0195	0.0190	0.0185	0.0194	2.51	1.21E-02	9741.76	9516.93	9971.8
CGGGGA	73	138	0.0002	0.0001	0.0001	0.0002	2.50	1.26E-02	50.973	36.8516	70.5047
GTGCA	1389	3475	0.0032	0.0030	0.0028	0.0032	2.49	1.27E-02	1283.49	1202.92	1369.44
CTTGTC	535	1274	0.0014	0.0012	0.0011	0.0014	2.49	1.27E-02	470.633	422.823	523.841
GGGC	2801	7181	0.0054	0.0052	0.0049	0.0054	2.45	1.43E-02	2652.65	2535.79	2774.86
CAGG	5452	14203	0.0106	0.0102	0.0099	0.0105	2.42	1.54E-02	5246.56	5081.55	5416.87
GTGAAC	400	938	0.0012	0.0010	0.0009	0.0011	2.41	1.60E-02	346.469	305.806	392.533
GTCTTG	570	1369	0.0015	0.0013	0.0012	0.0015	2.40	1.63E-02	505.727	456.075	560.776
GAAAGGG	192	422	0.0006	0.0005	0.0004	0.0005	2.40	1.64E-02	155.857	129.399	187.722
GGGAA	2308	5893	0.0054	0.0051	0.0048	0.0054	2.39	1.67E-02	2176.57	2070.96	2287.54
TGGGA	2322	5931	0.0054	0.0051	0.0049	0.0054	2.39	1.70E-02	2190.61	2084.65	2301.92
TGGGT	1976	5024	0.0038	0.0036	0.0034	0.0038	2.37	1.78E-02	1855.72	1758.34	1958.47
TGCTG	2911	7486	0.0043	0.0040	0.0039	0.0042	2.36	1.81E-02	2764.84	2645.42	2889.63
GCAGAG	456	1082	0.0012	0.0010	0.0009	0.0012	2.36	1.81E-02	399.705	355.842	448.969
TCTTGTC	199	441	0.0005	0.0004	0.0004	0.0005	2.35	1.88E-02	162.885	135.782	195.395
ATGGG	1610	4069	0.0038	0.0035	0.0033	0.0037	2.34	1.91E-02	1502.88	1415.5	1595.63
CCTGTGC	139	296	0.0004	0.0003	0.0003	0.0004	2.34	1.92E-02	109.322	87.5565	136.495

GTGTGTG	885	2184	0.0009	0.0009	0.0008	0.0009	2.33	1.98E-02	806.574	743.18	875.371
GGAG	5562	14516	0.0081	0.0078	0.0076	0.0081	2.33	1.98E-02	5362.1	5195.06	5534.46
CTGTGC	562	1354	0.0015	0.0013	0.0012	0.0014	2.33	2.01E-02	500.186	450.82	554.949
AAAGGGG	160	348	0.0005	0.0004	0.0004	0.0005	2.30	2.15E-02	128.518	104.716	157.727
ATGGGT	452	1076	0.0013	0.0012	0.0010	0.0013	2.30	2.16E-02	397.442	353.712	446.571
TTGGGG	644	1566	0.0019	0.0017	0.0015	0.0019	2.30	2.16E-02	578.433	525.163	637.097
GTGGG	1526	3855	0.0030	0.0028	0.0026	0.0029	2.29	2.19E-02	1423.92	1338.91	1514.32
AGAGTTC	146	315	0.0005	0.0004	0.0003	0.0005	2.27	2.29E-02	116.331	93.8032	144.266
GGTGT	1585	4012	0.0037	0.0035	0.0033	0.0037	2.27	2.30E-02	1481.83	1395.08	1573.95
TTGCAAG	167	366	0.0006	0.0005	0.0004	0.0006	2.27	2.32E-02	135.165	110.694	165.044
GGGGAT	340	795	0.0010	0.0009	0.0007	0.0010	2.26	2.35E-02	293.649	256.412	336.289
TCTGG	2232	5712	0.0052	0.0049	0.0047	0.0052	2.26	2.36E-02	2109.72	2005.78	2219.02
GGATTAG	95	194	0.0003	0.0002	0.0002	0.0003	2.26	2.38E-02	71.65	54.4804	94.2292
AGTGCTG	175	386	0.0006	0.0005	0.0004	0.0006	2.25	2.41E-02	142.551	117.355	173.155
GAATTGC	142	306	0.0005	0.0004	0.0003	0.0005	2.25	2.42E-02	113.007	90.8383	140.584
CTGGTA	422	1003	0.0012	0.0011	0.0010	0.0012	2.25	2.46E-02	370.478	328.344	418.012
TTGGGA	744	1828	0.0022	0.0020	0.0018	0.0022	2.23	2.55E-02	675.208	617.454	738.353
CTTGGGG	142	307	0.0005	0.0004	0.0003	0.0005	2.22	2.62E-02	113.376	91.1674	140.993
CTTGG	2156	5520	0.0050	0.0048	0.0045	0.0050	2.21	2.74E-02	2038.8	1936.66	2146.31
TCAGGGG	82	165	0.0003	0.0002	0.0002	0.0003	2.21	2.74E-02	60.9352	45.282	81.998
TGTGTGT	1170	2936	0.0012	0.0012	0.0011	0.0012	2.20	2.77E-02	1084.3	1010.39	1163.6
TGAGTG	665	1628	0.0013	0.0012	0.0011	0.0013	2.19	2.84E-02	601.258	546.884	661.031
GAGGGC	224	509	0.0007	0.0005	0.0005	0.0007	2.19	2.87E-02	188.009	158.709	222.716
TGGATGG	165	364	0.0004	0.0003	0.0002	0.0003	2.19	2.88E-02	134.448	110.047	164.258
GGGGGA	349	822	0.0010	0.0009	0.0008	0.0010	2.18	2.90E-02	303.622	265.715	346.931
CAGGTGT	129	277	0.0004	0.0003	0.0003	0.0004	2.18	2.92E-02	102.297	81.3215	128.681
GAACC	913	2271	0.0021	0.0020	0.0018	0.0021	2.17	3.03E-02	838.791	774.123	908.849
AGGGA	1936	4947	0.0038	0.0036	0.0034	0.0038	2.16	3.08E-02	1827.28	1730.66	1929.27
GATGG	1589	4036	0.0031	0.0029	0.0027	0.0031	2.16	3.09E-02	1490.78	1403.74	1583.2
ATGCCC	288	670	0.0008	0.0007	0.0006	0.0008	2.15	3.12E-02	247.478	213.497	286.863
CTGT	12016	31796	0.0234	0.0229	0.0224	0.0233	2.15	3.14E-02	11745.4	11498.7	11997.2
TGGGCA	424	1014	0.0012	0.0011	0.0010	0.0012	2.15	3.18E-02	374.541	332.163	422.319
GTGGGG	402	958	0.0010	0.0009	0.0008	0.0010	2.15	3.18E-02	353.898	312.772	400.425
AGAACC	356	842	0.0010	0.0009	0.0008	0.0010	2.14	3.24E-02	311.009	272.614	354.807
GTATCT	629	1540	0.0018	0.0017	0.0015	0.0018	2.13	3.34E-02	568.83	516.024	627.029
AGTGGG	439	1054	0.0013	0.0011	0.0010	0.0013	2.12	3.43E-02	389.316	346.061	437.97
GGTGGA	369	877	0.0011	0.0009	0.0008	0.0011	2.10	3.56E-02	323.937	284.701	368.575
GGTC	3101	8032	0.0060	0.0058	0.0055	0.0060	2.10	3.61E-02	2967	2843.3	3096.06

GGATG	1594	4058	0.0031	0.0029	0.0027	0.0031	2.08	3.71E-02	1498.91	1411.62	1591.57
TTGAGG	591	1447	0.0017	0.0016	0.0014	0.0017	2.06	3.92E-02	534.478	483.368	590.983
