# Supplementary material for: Identification of motifs that function in the splicing of non-canonical introns
Source: Genome Biol. 2008 Jun 12;9(6):R97. doi: 10.1186/gb-2008-9-6-r97 (PMC2481429; doi:10.1186/gb-2008-9-6-r97)
Supplement: Additional data file 4 — Listing of all clusters derived from n-mers enriched in the 50 nucleotide region upstream of weak PY tracts from GC-rich introns. Included are the individual n-mers and associated statistics used to produce each motif. [file gb-2008-9-6-r97-S4.pdf]

Additional Table 4: N-mer clusters derived from n-mers enriched upstream of GC-rich introns

**Field**	**Description**
ClustID	ID assigned to cluster
Nmer	Individual n-mer
GCS	Greatest common substring shared between members of cluster
Aligned	Alignment of n-mers according to GCS
Count	Occurrences of n-mer in orginal sample
Zscore	Z-score for enrichment of n-mer in original sample

clustID	Nmer	GCS	Aligned	Count	Zscore
GC01	GCCCC	GCCCC	'--GCCCC-	9070	3.31
	GCCCCC	GCCCC	'--GCCCCC	2438	2.43
	GCCCCG	GCCCC	'--GCCCCG	1269	2.81
	GGCCCC	GCCCC	'-GGCCCC-	3015	2.26
	GGCCCCG	GCCCC	'-GGCCCCG	476	2.02
	GTGCCCC	GCCCC	'GTGCCCC-	549	1.92
GC02	AGGGGG	GGGGG	'-AGGGGG-	1925	2.64
	CCGGGGG	GGGGG	'CCGGGGG-	331	1.87
	CGGGGG	GGGGG	'-CGGGGG-	1191	2.96
	GAGGGGG	GGGGG	'GAGGGGG-	781	2.22
	GGGGG	GGGGG	'--GGGGG-	8965	6.28
	GGGGGA	GGGGG	'--GGGGGA	1885	2.12
	GGGGGG	GGGGG	'--GGGGGG	1769	3.04
	GGGGGT	GGGGG	'--GGGGGT	2208	3.06
GC03	AGGGAGG	GGGAGG	'AGGGAGG-	1345	2.05
	GGGAGG	GGGAGG	'-GGGAGG-	4992	4.47
	GGGAGGC	GGGAGG	'-GGGAGGC	1034	2.45
	GGGAGGG	GGGAGG	'-GGGAGGG	2178	3.26
	TGGGAGG	GGGAGG	'TGGGAGG-	1311	2.09
GC04	AGGTGGG	GGTGGG	'AGGTGGG-	1164	1.95
	GGGTGGG	GGTGGG	'GGGTGGG-	2534	4.52
	GGTGGG	GGTGGG	'-GGTGGG-	5025	5.36
	GGTGGGC	GGTGGG	'-GGTGGGC	1071	2.35
	GGTGGGT	GGTGGG	'-GGTGGGT	818	2.43
GC05	GGGCGG	GGGCG	'-GGGCGG-	1694	3.86
	GGGCGGG	GGGCG	'-GGGCGGG	955	3.19
	GGGGCG	GGGCG	'GGGGCG--	1384	2.91
	GGGGCGG	GGGCG	'GGGGCGG-	743	2.36
	TGGGCG	GGGCG	'TGGGCG--	803	2.52
	TGGGCGG	GGGCG	'TGGGCGG-	372	2.16
	GGCTG	GGCTG	'-GGCTG--	9896	3.06
	GGCTGCG	GGCTG	'-GGCTGCG	312	1.91
	GGCTGG	GGCTG	'-GGCTGG-	4136	2.77

GC06	GGCTGGG	GGCTG	'-GGCTGGG	2117	2.50
	GGCTGTG	GGCTG	'-GGCTGTG	1006	2.16
	GGGCTG	GGCTG	'GGGCTG--	4356	3.17
	GGGCTGG	GGCTG	'GGGCTGG-	1949	2.25
GC07	AGGCAGG	GGCAG	'-AGGCAGG-	1048	2.12
	AGGGCAG	GGCAG	'AGGGCAG--	1125	1.97
	GGCAGG	GGCAG	'--GGCAGG-	3961	3.94
	GGCAGGC	GGCAG	'--GGCAGGC	836	1.90
	GGGCAG	GGCAG	'-GGGCAG--	4115	3.90
	GGGCAGG	GGCAG	'-GGGCAGG-	1928	3.36
	GGGGCAG	GGCAG	'GGGGCAG--	1396	2.21
	TGGGCAG	GGCAG	'TGGGCAG--	1145	1.99
GC08	CCCGGGG	CCGGG	'CCCGGGG-	480	1.97
	CCGGG	CCGGG	'-CCGGG--	3641	4.73
	CCGGGA	CCGGG	'-CCGGGA-	675	2.09
	CCGGGC	CCGGG	'-CCGGGC-	1022	1.84
	CCGGGG	CCGGG	'-CCGGGG-	1298	3.47
	CCGGGGA	CCGGG	'-CCGGGGA	273	1.89
	GCCGGGG	CCGGG	'GCCGGGG-	479	2.37
GC09	AGGGGTG	GGGGTG	'AGGGGTG-	773	1.83
	CGGGGTG	GGGGTG	'CGGGGTG-	351	1.91
	GGGGGTG	GGGGTG	'GGGGGTG-	1180	2.53
	GGGGTG	GGGGTG	'-GGGGTG-	3701	4.78
	GGGGTGC	GGGGTG	'-GGGGTGC	692	2.58
	GGGGTGG	GGGGTG	'-GGGGTGG	1932	4.01
	TGGGGTG	GGGGTG	'TGGGGTG-	1285	3.12
GC10	GGTGGGG	GTGGGG	'GGTGGGG-	2137	3.72
	GTGGGG	GTGGGG	'-GTGGGG-	4312	4.75
	GTGGGGA	GTGGGG	'-GTGGGGA	996	1.82
	GTGGGGC	GTGGGG	'-GTGGGGC	1098	2.17
	GTGGGGG	GTGGGG	'-GTGGGGG	1347	3.01
	GTGGGGT	GTGGGG	'-GTGGGGT	809	2.43
	TGTGGGG	GTGGGG	'TGTGGGG-	965	1.90
GC11	CGGGGGC	GGGGGC	'CGGGGGC-	407	1.92
	GGGGGC	GGGGGC	'-GGGGGC-	2964	3.97
	GGGGGCC	GGGGGC	'-GGGGGCC	783	1.84
	GGGGGCG	GGGGGC	'-GGGGGCG	476	2.07
	TGGGGGC	GGGGGC	'TGGGGGC-	1206	2.49
	ATGGGG	TGGG	'-ATGGGG--	1705	2.14
	CCTGGGG	TGGG	'CCTGGGG--	1738	2.08
	CTGGG	TGGG	'-CTGGG---	13703	4.55
	CTGGGA	TGGG	'-CTGGGA--	2631	1.82
	CTGGGC	TGGG	'-CTGGGC--	3698	2.72

	CTGGGG	TGGG	'-CTGGGG--	4867	3.04
	CTGGGGT	TGGG	'-CTGGGGT-	1105	2.22
	GCTGGG	TGGG	'GCTGGG---	4398	3.38
GC12	GCTGGGC	TGGG	'GCTGGGC--	1140	2.26
	GCTGGGG	TGGG	'GCTGGGG--	1680	2.18
	TGGGG	TGGG	'--TGGGG--	13314	6.31
	TGGGGA	TGGG	'--TGGGGA-	3033	1.95
	TGGGGC	TGGG	'--TGGGGC-	3452	2.89
	TGGGGG	TGGG	'--TGGGGG-	3851	3.79
	TGGGGGG	TGGG	'--TGGGGGG	804	1.89
	TGGGGGT	TGGG	'--TGGGGGT	984	2.02
	TGGGGT	TGGG	'--TGGGGT-	2774	3.69
	TGGGGTC	TGGG	'--TGGGGTC	763	1.91
	AGGGGC	GGGGC	'-AGGGGC--	2690	2.44
	AGGGGCA	GGGGC	'-AGGGGCA-	675	1.95
	CGGGGC	GGGGC	'-CGGGGC--	1382	3.21
	CGGGGCC	GGGGC	'-CGGGGCC-	503	1.90
	GAGGGGC	GGGGC	'GAGGGGC--	1079	2.17
	GGGGC	GGGGC	'--GGGGC--	10751	6.27
GC13	GGGGCA	GGGGC	'--GGGGCA-	2491	2.63
	GGGGCC	GGGGC	'--GGGGCC-	3271	3.01
	GGGGCCG	GGGGC	'--GGGGCCG	506	2.24
	GGGGCT	GGGGC	'--GGGGCT-	3377	3.63
	GGGGCTG	GGGGC	'--GGGGCTG	1810	2.95
	GGGGGCT	GGGGC	'-GGGGGCT-	916	2.27
	TGGGGCT	GGGGC	'-TGGGGCT-	1149	1.93
	AGGGAG	GGGAG	'-AGGGAG--	2733	2.16
	AGGGGAG	GGGAG	'AGGGGAG--	926	1.95
	CGGGAG	GGGAG	'-CGGGAG--	874	2.08
	CTGGGAG	GGGAG	'CTGGGAG--	1169	2.29
	GGGAG	GGGAG	'--GGGAG--	10466	5.34
GC14	GGGAGC	GGGAG	'--GGGAGC-	2305	2.78
	GGGAGCC	GGGAG	'--GGGAGCC	848	1.84
	GGGGAG	GGGAG	'-GGGGAG--	3598	3.49
	GGGGAGG	GGGAG	'-GGGGAGG-	1786	2.83
	TGGGAG	GGGAG	'-TGGGAG--	2968	2.71
	TGGGGAG	GGGAG	'TGGGGAG--	1346	1.88
	CGCGGG	GCGGG	'CGCGGG--	442	2.27
	GCGGG	GCGGG	'-GCGGG--	3687	5.61
	GCGGGC	GCGGG	'-GCGGGC-	850	3.02
	GCGGGCG	GCGGG	'-GCGGGCG	214	2.10
	GCGGGG	GCGGG	'-GCGGGG-	1578	3.70
GC15	GCGGGGC	GCGGG	'-GCGGGGC	576	2.55

GC15	GCGGGGG	GCGGG	'-GCGGGGG	450	1.90
	GCGGGT	GCGGG	'-GCGGGT-	558	2.48
	GGCGGG	GCGGG	'GGCGGG--	1832	4.42
	GGCGGGC	GCGGG	'GGCGGGC-	420	2.29
	GGCGGGG	GCGGG	'GGCGGGG-	833	2.89
	TGCGGG	GCGGG	'TGCGGG--	692	2.33
GC16	CGGGTG	GGTG	'-CGGGTG--	793	2.36
	GCGGGTG	GGTG	'GCGGGTG--	301	1.90
	GGGTG	GGTG	'--GGGTG--	9729	6.17
	GGGTGC	GGTG	'--GGGTGC-	1922	3.33
	GGGTGCA	GGTG	'--GGGTGCA	484	2.40
	GGGTGG	GGTG	'--GGGTGG-	4768	5.15
	GGTGC	GGTG	'---GGTGC-	4477	3.53
	GGTGCA	GGTG	'---GGTGCA	989	2.06
	GGTGCC	GGTG	'---GGTGCC	1643	2.03
	GGTGG	GGTG	'---GGTGG-	9986	5.48
	GTGGGTG	GGTG	'GTGGGTG--	1010	2.71
	TGGGTG	GGTG	'-TGGGTG--	2786	2.84
	TGGGTGG	GGTG	'-TGGGTGG-	1253	2.41
GC17	AGAGGG	GAGG	'-AGAGGG-	2022	1.96
	GAGGG	GAGG	'--GAGGG-	9317	4.71
	GAGGGC	GAGG	'--GAGGGC	2158	2.65
	GAGGGG	GAGG	'--GAGGGG	3255	3.56
	GGAGG	GAGG	'-GGAGG--	10852	5.10
	GGAGGA	GAGG	'-GGAGGA-	2176	2.00
	GGAGGC	GAGG	'-GGAGGC-	2497	2.54
	GGAGGCA	GAGG	'-GGAGGCA	663	2.22
	GGAGGG	GAGG	'-GGAGGG-	4418	3.79
	GGAGGGC	GAGG	'-GGAGGGC	968	2.01
	GGAGGGG	GAGG	'-GGAGGGG	1641	2.65
	TGGAGG	GAGG	'TGGAGG--	2409	1.94
GC18	CCCCCGG	CCCGG	'CCCCCGG-	323	2.05
	CCCCGG	CCCGG	'-CCCCGG-	1248	3.15
	CCCCGGG	CCCGG	'-CCCCGGG	527	2.38
	CCCGG	CCCGG	'--CCCGG-	3359	4.10
	CCCGGC	CCCGG	'--CCCGGC	1016	1.98
	CCCGGG	CCCGG	'--CCCGGG	1425	3.09
	GCCCCGG	CCCGG	'GCCCCGG-	431	2.10
	GCCCGG	CCCGG	'-GCCCGG-	936	1.89
	AGGCCG	GCCG	'AGGCCG--	619	1.98
	CGGCCG	GCCG	'CGGCCG--	373	2.02
	GCCGC	GCCG	'--GCCGC-	1826	2.06
	GCCGG	GCCG	'--GCCGG-	2534	3.17

GC19	GCCGGG	GCCG	'--GCCGGG	1251	3.29
	GGCCG	GCCG	'-GGCCG--	2938	4.30
	GGCCGC	GCCG	'-GGCCGC-	775	2.11
	GGCCGG	GCCG	'-GGCCGG-	1230	3.07
	GGCCGGG	GCCG	'-GGCCGGG	655	2.96
GC20	AGGGCG	GGCG	'AGGGCG--	690	2.14
	CGGGCG	GGCG	'CGGGCG--	436	2.23
	GGCGC	GGCG	'--GGCGC-	1718	2.37
	GGCGCG	GGCG	'--GGCGCG	348	2.12
	GGGCG	GGCG	'-GGGCG--	3407	5.03
	GGGCGC	GGCG	'-GGGCGC-	741	2.26
	GGGCGCG	GGCG	'-GGGCGCG	177	1.89
GC21	CGTGGG	GTGGG	'CGTGGG--	951	2.38
	GTGGG	GTGGG	'-GTGGG--	10631	6.19
	GTGGGC	GTGGG	'-GTGGGC-	2358	2.38
	GTGGGCG	GTGGG	'-GTGGGCG	338	2.07
	GTGGGT	GTGGG	'-GTGGGT-	1837	2.83
	TGTGGG	GTGGG	'TGTGGG--	2499	2.02
GC22	CAGGG	CAGGG	'--CAGGG-	10084	3.95
	CAGGGC	CAGGG	'--CAGGGC	3154	2.11
	CAGGGG	CAGGG	'--CAGGGG	2747	3.11
	GCAGGG	CAGGG	'-GCAGGG-	3505	3.46
	GCAGGGG	CAGGG	'-GCAGGGG	1110	2.49
	GGCAGGG	CAGGG	'GGCAGGG-	1699	2.94
GC23	CCCCGCC	CCGCC	'CCCCGCC-	657	2.01
	CCCGCC	CCGCC	'-CCCGCC-	1298	2.28
	CCCGCCC	CCGCC	'-CCCGCCC	626	2.06
	CCGCC	CCGCC	'--CCGCC-	2671	2.38
	CCGCCC	CCGCC	'--CCGCCC	1173	2.02
GC24	CCTGG	CCTGG	'--CCTGG-	11975	3.13
	CCTGGG	CCTGG	'--CCTGGG	5096	3.20
	GCCTGG	CCTGG	'-GCCTGG-	3678	2.98
	GCCTGGG	CCTGG	'-GCCTGGG	1566	2.73
	GGCCTGG	CCTGG	'GGCCTGG-	1614	2.68
GC25	CGGGCC	GGGCC	'CGGGCC--	850	1.98
	GGGCC	GGGCC	'-GGGCC--	9235	4.55
	GGGCCC	GGGCC	'-GGGCCC-	2982	2.85
	GGGCCG	GGGCC	'-GGGCCG-	1257	3.06
	GGGCCGG	GGGCC	'-GGGCCGG	595	2.64
	GGGCCTG	GGGCC	'-GGGCCTG	1281	1.88
	TGGGCC	GGGCC	'TGGGCC--	2592	2.20
	TGGGCCC	GGGCC	'TGGGCCC-	923	2.03
