# Supplementary material for: Identification of motifs that function in the splicing of non-canonical introns
Source: Genome Biol. 2008 Jun 12;9(6):R97. doi: 10.1186/gb-2008-9-6-r97 (PMC2481429; doi:10.1186/gb-2008-9-6-r97)
Supplement: Additional data file 5 — Listing of all clusters derived from n-mers enriched in the 50 nucleotide region upstream of weak PY tracts from AT-rich introns. Included are the individual n-mers and associated statistics used to produce each motif. [file gb-2008-9-6-r97-S5.pdf]

Additional Table 5: N-mer clusters derived from n-mers enriched upstream of AT-rich introns

**Field**	**Description**
ClustID	ID assigned to cluster
Nmer	Individual n-mer
GCS	Greatest common substring shared between members of cluster
Aligned	Alignment of n-mers according to GCS
Count	Occurrences of n-mer in orginal sample
Zscore	Z-score for enrichment of n-mer in original sample

clustID	Nmer	GCS	Aligned	Count	Zscore
AT01	AAAGGGG	GGGG	'AAAGGGG--	160	2.30
	AAGGGG	GGGG	'-AAGGGG--	393	2.68
	AGGGG	GGGG	'--AGGGG--	1188	2.84
	CGGGGA	GGGG	'--CGGGGA-	73	2.50
	CTTGGGG	GGGG	'CTTGGGG--	142	2.22
	GGGG	GGGG	'---GGGG--	4240	5.44
	GGGGA	GGGG	'---GGGGA-	1508	3.05
	GGGGAT	GGGG	'---GGGGAT	340	2.26
	GGGGG	GGGG	'---GGGGG-	924	3.25
	GGGGGA	GGGG	'---GGGGGA	349	2.18
	GGGGGT	GGGG	'---GGGGGT	256	2.58
	GGGGT	GGGG	'---GGGGT-	1092	2.78
	GTGGGG	GGGG	'-GTGGGG--	402	2.15
	TCAGGGG	GGGG	'TCAGGGG--	82	2.21
	TGGGG	GGGG	'--TGGGG--	1858	3.28
	TGGGGG	GGGG	'--TGGGGG-	452	2.98
	TGGGGGT	GGGG	'--TGGGGGT	136	2.76
	TTGGGG	GGGG	'-TTGGGG--	644	2.30
AT02	AGTGGG	TGGG	'AGTGGG--	439	2.12
	ATGGG	TGGG	'-ATGGG--	1610	2.34
	ATGGGT	TGGG	'-ATGGGT-	452	2.30
	GTGGG	TGGG	'-GTGGG--	1526	2.29
	TGGG	TGGG	'--TGGG--	7385	5.03
	TGGGA	TGGG	'--TGGGA-	2322	2.39
	TGGGCA	TGGG	'--TGGGCA	424	2.15
	TGGGT	TGGG	'--TGGGT-	1976	2.37
	TTGGG	TGGG	'-TTGGG--	2360	3.44
	TTGGGA	TGGG	'-TTGGGA-	744	2.23
AT03	CCTGTG	TGTG	'CCTGTG---	689	2.60
	CTGTG	TGTG	'-CTGTG---	3315	2.90
	GTGTG	TGTG	'-GTGTG---	3251	3.16
	GTGTGT	TGTG	'-GTGTGT--	1697	2.88
	GTGTGTG	TGTG	'-GTGTGTG-	885	2.33

	TGTG	TGTG	'--TGTG---	14356	4.28
	TGTGT	TGTG	'--TGTGT--	5752	2.93
	TGTGTG	TGTG	'--TGTGTG-	1949	2.91
	TGTGTGT	TGTG	'--TGTGTGT	1170	2.20
AT04	CTTG	CTTG	'--CTTG---	9573	2.66
	CTTGAGG	CTTG	'--CTTGAGG	135	2.62
	CTTGG	CTTG	'--CTTGG--	2156	2.21
	CTTGTC	CTTG	'--CTTGTC-	535	2.49
	GTCTTG	CTTG	'GTCTTG---	570	2.40
	TCTTGTC	CTTG	'-TCTTGTC-	199	2.35
AT05	CCTGTGC	GTGC	'CCTGTGC-	139	2.34
	CTGTGC	GTGC	'-CTGTGC-	562	2.33
	GTGC	GTGC	'---GTGC-	4546	3.07
	GTGCA	GTGC	'---GTGCA	1389	2.49
	TGTGC	GTGC	'--TGTGC-	2137	2.97
	TGTGCA	GTGC	'--TGTGCA	704	2.96
AT06	AGGGA	GGA	'AGGGA----	1936	2.16
	GGATG	GGA	'--GGATG--	1594	2.08
	GGATTAG	GGA	'--GGATTAG	95	2.26
	GGGA	GGA	'-GGGA----	6088	4.32
	GGGAA	GGA	'-GGGAA---	2308	2.39
	GGGAT	GGA	'-GGGAT---	1367	2.65
	TGGATGG	GGA	'-TGGATGG-	165	2.19
AT07	AGAGTTC	GAG	'--AGAGTTC	146	2.27
	GAGG	GAG	'---GAGG--	5356	2.75
	GAGGG	GAG	'---GAGGG-	1316	2.63
	GAGGGC	GAG	'---GAGGGC	224	2.19
	GAGT	GAG	'---GAGT--	6647	2.68
	GCAGAG	GAG	'GCAGAG---	456	2.36
	GGAG	GAG	'--GGAG---	5562	2.33
	TGAG	GAG	'--TGAG---	9029	3.01
	TGAGG	GAG	'--TGAGG--	1897	2.70
	TGAGTG	GAG	'--TGAGTG-	665	2.19
	TTGAGG	GAG	'-TTGAGG--	591	2.06
AT08	CTGGTA	TGG	'-CTGGTA	422	2.25
	GATGG	TGG	'GATGG--	1589	2.16
	GGTGGA	TGG	'GGTGGA-	369	2.10
	TCTGG	TGG	'TCTGG--	2232	2.26
	TTGG	TGG	'-TTGG--	10030	2.51
