# Supplementary figures and images for: Identification of motifs that function in the splicing of non-canonical introns
Source: Genome Biol. 2008 Jun 12;9(6):R97. doi: 10.1186/gb-2008-9-6-r97 (PMC2481429; doi:10.1186/gb-2008-9-6-r97)

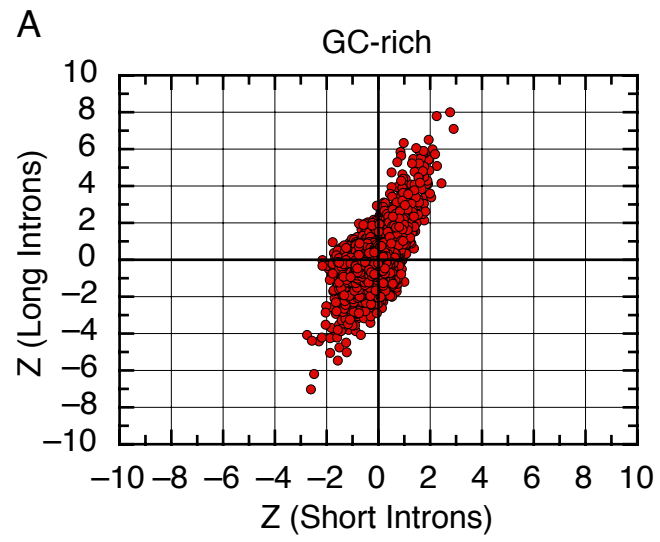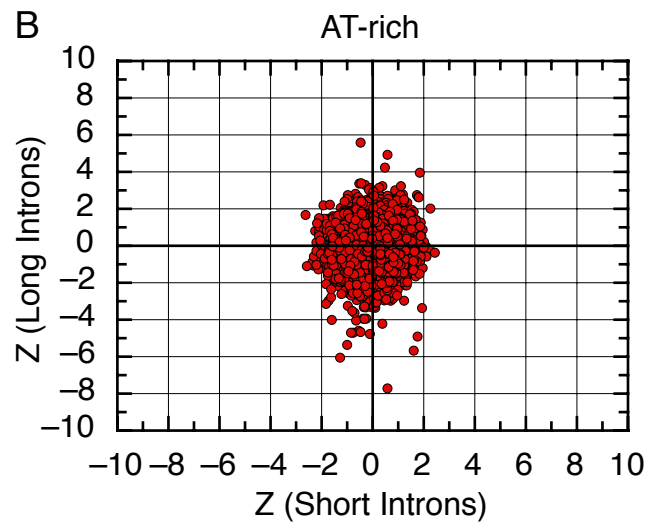

Supplement: Additional data file 6 — The Z-scores for enrichment of all 4-7 nucleotide n-mers in the intronic region upstream (-80 to -30 relative to the acceptor splice-junction) of PY tracts with low S65 scores for short (<200 nucleotide) introns is plotted versus long (≥ 200 nucleotide) introns. (a) Data for GC-rich introns. (b) Data for AT-rich introns. [file gb-2008-9-6-r97-S6.pdf]

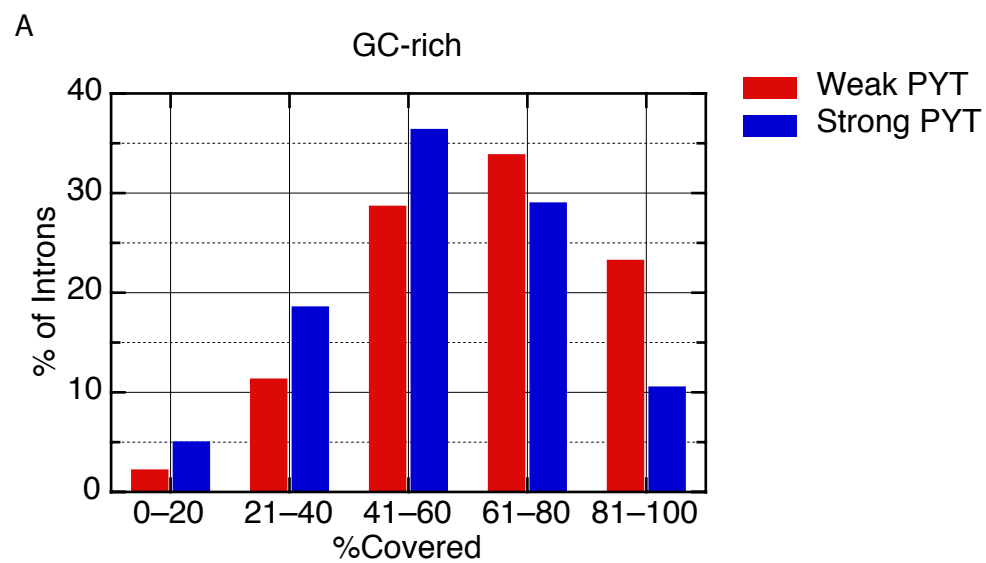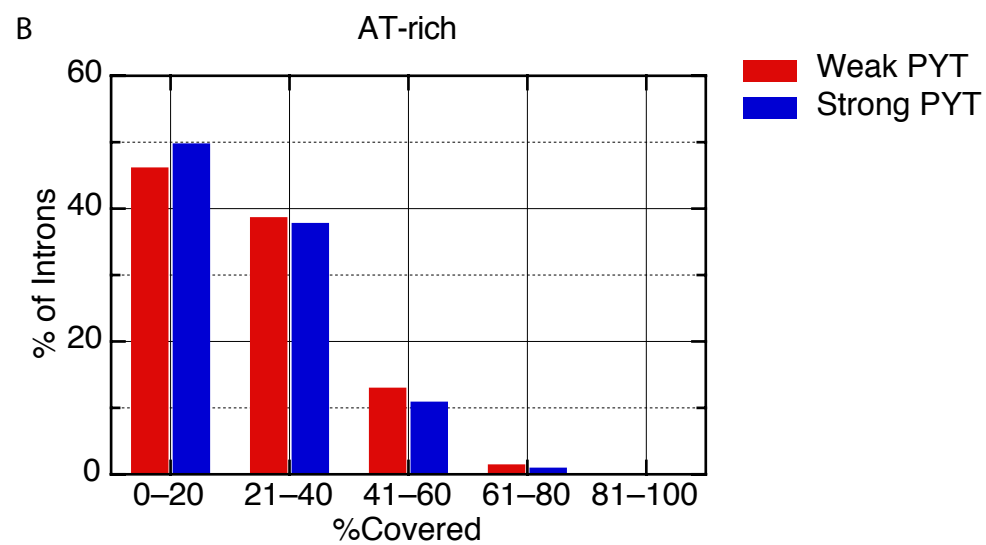

Supplement: Additional data file 7 — The portion of the sequence corresponding to the -80 to -30 region matching one or more of the n-mers enriched in the same region for introns with weak PY tracts (Additional data files 2 and 3) was determined. These values (referred to as the percent coverage) were binned as indicated along the x-axis. [file gb-2008-9-6-r97-S7.pdf]
